# Both entry to and exit from diapause arrest in *Caenorhabditis elegans* are regulated by a steroid hormone pathway

**DOI:** 10.1242/dev.200173

**Published:** 2022-05-03

**Authors:** Mark G. Zhang, Paul W. Sternberg

**Affiliations:** Division of Biology and Biological Engineering, California Institute of Technology, Pasadena, CA 91125, USA

**Keywords:** Dauer, Diapause, Hormone, Decision, *daf-9*, *daf-12*

## Abstract

Diapause arrest in animals such as *Caenorhabditis elegans* is tightly regulated so that animals make appropriate developmental decisions amidst environmental challenges. Fully understanding diapause requires mechanistic insight of both entry and exit from the arrested state. Although a steroid hormone pathway regulates the entry decision into *C. elegans* dauer diapause, its role in the exit decision is less clear. A complication to understanding steroid hormonal regulation of dauer has been the peculiar fact that steroid hormone mutants such as *daf-9* form partial dauers under normal growth conditions. Here, we corroborate previous findings that *daf-9* mutants remain capable of forming full dauers under unfavorable growth conditions and establish that the *daf-9* partial dauer state is likely a partially exited dauer that has initiated but cannot complete the dauer exit process. We show that the steroid hormone pathway is both necessary for and promotes complete dauer exit, and that the spatiotemporal dynamics of steroid hormone regulation during dauer exit resembles that of dauer entry. Overall, dauer entry and dauer exit are distinct developmental decisions that are both controlled by steroid hormone signaling.

## Abstract (precis)

**Summary:** In animals such as *Caenorhabditis elegans*, a steroid hormone pathway controls both the entry and exit decisions into and out of the developmentally arrested dauer state in response to environmental signaling.

## INTRODUCTION

Animals must be able to adapt to changing environments to survive against uncertain and stress-inducing circumstances. One such adaptive mechanism is diapause, a state of developmental arrest typically characterized by metabolic depression and stress resistance (Hand et al., 2016). Diapause is a dynamic process that involves successive developmental decisions dictating entry, maintenance and exit from the dormant state (Koštál, 2006; Ragland et al., 2010). Diapause is well conserved across the animal kingdom including nematodes, insects, crustaceans, fish and mammals (Hand et al., 2016; Hu, 2007; Podrabsky and Hand, 2015; Ragland et al., 2010).

Upon encountering adverse conditions during larval growth, *Caenorhabditis elegans* exit the cycle of reproductive development and instead enter the alternative, diapause state, termed dauer, granting them increased durability and longevity to protect against environmental insults, allowing them to disperse in search of a more favorable environment (Cassada and Russell, 1975; Hu, 2007). The dauer entry decision-making process comprises two distinct subdecisions. First-stage (L1) larvae decide between developing into L2 or pre-dauer L2d larvae, depending on whether conditions are favorable or unfavorable, respectively (the ‘L1 to L2/L2d subdecision’). If conditions sufficiently improve, then L2d larvae choose reproductive development by becoming L3 larvae but, if not, they become dauer larvae (the ‘L2d to L3/Dauer subdecision’; Golden and Riddle, 1984). While in the dauer state, animals continuously assess their environment and, when conditions improve by way of an increased food to pheromone ratio, exit the dauer state to return to the reproductive cycle as L4 larvae (Golden and Riddle, 1982). A complete understanding of this developmental decision-making process requires a synthesis of information involving both the dauer entry subdecisions and the dauer exit decision. The majority of dauer studies in *C. elegans* have focused on dauer entry (Androwski et al., 2017; Fielenbach and Antebi, 2008; Hu, 2007), leaving much to be explored for dauer exit.

Previous studies have found multiple pathways that govern the dauer entry decision, including cGMP signaling, insulin growth factor signaling, TGF-β signaling and steroid hormone signaling (Fielenbach and Antebi, 2008; Hu, 2007). The steroid hormone pathway has been placed genetically downstream in the dauer entry process and is thought to serve as a convergence point for both the insulin and the TGF-β signaling pathways (Fielenbach and Antebi, 2008) in controlling dauer development. The steroid hormone pathway centers on DAF-12, a nuclear hormone receptor with homology to the vertebrate farnesoid-X receptor (FXR, also known as Nr1h4; Antebi, 2015; Antebi et al., 1998). The major endogenous ligands for DAF-12/FXR are steroid hormones collectively referred to as dafachronic acids (DA), which include Δ7-DA (dafa#2) and Δ4-DA (dafa#4) among others (Aguilaniu et al., 2016; Mahanti et al., 2014; Motola et al., 2006). DAF-12/FXR regulation of its transcriptional targets depends on environmental growth conditions, which in turn dictate the presence of DAF-12/FXR ligands. Under favorable conditions, DAF-9 catalyzes the formation of DAs such as Δ7-DA that bind to DAF-12/FXR and specify reproductive adulthood. Under unfavorable conditions, unliganded DAF-12/FXR interacts with the corepressor DIN-1/Cor to specify dauer entry (Fielenbach and Antebi, 2008).

Biosynthesis of all known DAs requires the cytochrome P450 enzyme DAF-9, and therefore *daf-9* null mutants are completely dauer formation constitutive (Daf-c) (Aguilaniu et al., 2016; Gerisch et al., 2001; Jia et al., 2002). *daf-9* is constitutively expressed in a pair of neuroendocrine cells termed the XXX cells but shows variable upregulation in the hypodermis depending on environmental conditions and developmental state (Gerisch et al., 2001; Schaedel et al., 2012). When larvae choose the reproductive pathway during either of the two dauer entry subdecisions, *daf-9* is upregulated throughout the hypodermis and amplifies steroid hormone production to instigate reproductive development (Gerisch et al., 2001; Schaedel et al., 2012). Although the role of the steroid hormone pathway in regulating dauer entry is well-characterized, how the same steroid hormone pathway governs the dauer exit decision remains substantially less clear.

A complete analysis concerning how steroid hormone signaling regulates dauer arrest must also account for the well-documented observation that Daf-c mutants impaired for steroid hormone biosynthesis and/or signaling such as *daf-9*, *daf-36* (encoding a Rieske-like oxygenase that catalyzes the first step of steroid hormone biosynthesis), *ncr-1* and *ncr-2* (encoding two putative cholesterol transporters), and Daf-c alleles of *daf-12* do not form full dauers under favorable growth conditions as do other Daf-c strains such as *daf-2(e1370)* or *daf-7(e1372)* (Antebi et al., 1998; Gerisch et al., 2001; Li et al., 2004; Rottiers et al., 2006). Full dauers are characterized by radial and pharyngeal constriction, immobility, pumping quiescence and a darkened intestine owing to increased fat storage (Cassada and Russell, 1975; Riddle and Albert, 1997). Steroid hormone mutants such as *daf-9* instead form ‘partial’ or ‘dauer-like’ larvae that resemble dauers but exhibit non-dauer traits such as sporadic pumping, increased mobility, a slightly enlarged pharynx and a lighter body (Albert and Riddle, 1988; Gerisch et al., 2001). Partial dauers are not exclusive to steroid hormone mutants, as they are also observed in double mutants involving *daf-16* (Ailion and Thomas, 2000; Vowels and Thomas, 1992), which encodes a homolog of the forkhead transcription factor FOXO and is the major downstream target of the insulin pathway (Ogg et al., 1997).

**Fig. 1.**
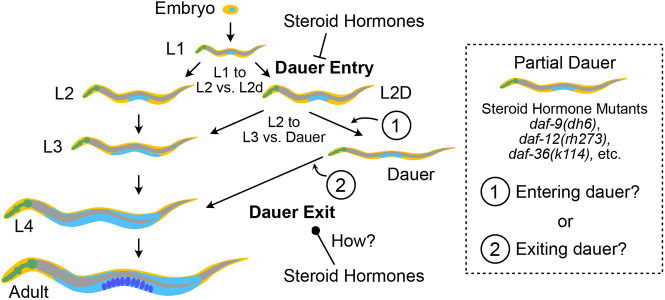
**Regulation of *C. elegans* dauer development by steroid hormones.** The *C. elegans* dauer pathway includes multiple developmental decisions. The dauer entry decision comprises two subdecisions made at L1 and then L2d, whereas dauer exit is a singular, continuous decision. Leftward or rightward arrows indicate the decision that is made under favorable or unfavorable conditions, respectively. Steroid hormones inhibit dauer entry and promote reproductive development. How steroid hormones regulate dauer exit is less understood. A model of how steroid hormones regulate the dauer pathway requires an understanding of why steroid hormone mutants such as *daf-9(dh6)*, *daf-12(rh273)* and *daf-36(k114)*, etc., form partial dauers rather than full dauers like other Daf-c mutants. Partial dauers could be (1) animals that have yet to enter a full dauer state or (2) partially exited dauers.

Whether steroid hormone mutants form partial or full dauers appears to depend on growth conditions, as *daf-9(dh6)* and *daf-12(rh273)* become full dauers under unfavorable growth conditions (Antebi et al., 1998; Gerisch et al., 2001). Why this distinction occurs remains unclear, but it has been speculated that the partial dauer may have first been a full dauer that attempted dauer exit (owing to favorable growth conditions) but could not complete it (Antebi et al., 1998). A comprehensive model of how steroid hormones govern dauer entry and exit should be able to explain why steroid hormone mutants form partial dauers under favorable growth conditions unlike other Daf-c strains (Fig. 1).

Here, we provide evidence that *daf-9* partial dauers are likely animals in a state of incomplete dauer exit rather than a state of incomplete dauer formation. Furthermore, we show that the *daf-9* partial dauer state requires insulin and TGF-β signaling*,* and that activation of these pathways is sufficient to induce a partial dauer state. We characterize the regulatory role of steroid hormones in dauer exit, demonstrating that the steroid hormone biosynthesis pathway is both necessary for and sufficient to induce dauer exit. We also show that the spatiotemporal regulation of *daf-9* during dauer exit closely mirrors that of the L1 to L2 versus L2d dauer entry subdecision, which means that *C. elegans* uses steroid hormone signaling in similar ways to regulate both the dauer entry and exit developmental decisions.

## RESULTS

### *daf-9* mutants form full dauers under unfavorable growth conditions

**Fig. 2.**
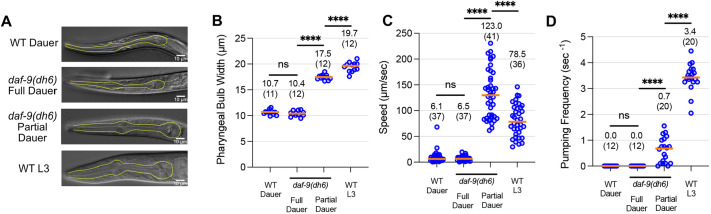
**Characterization of full versus partial dauers formed by *daf-9* null mutants.** (A-D) *daf-9(dh6)* full dauers formed under unfavorable growth conditions phenocopy wild-type dauers and are distinct from *daf-9(dh6)* partial dauers formed under favorable growth conditions. Phenotypes measured include terminal pharyngeal bulb width (A,B), speed (C) and pumping frequency (D). Wild-type L3 animals are shown for comparison. The pumping frequencies for WT and *daf-9(dh6)* full dauers in B are from the same experiment shown in Fig. 3F. Yellow outlines indicate pharynxes. ns, not significant. ****, *P*<0.0001 (Mann–Whitney test). Horizontal bars and in-graph numbers show median values. Numbers in parentheses indicate number of animals. Each dot is one animal.

**Fig. 3.**
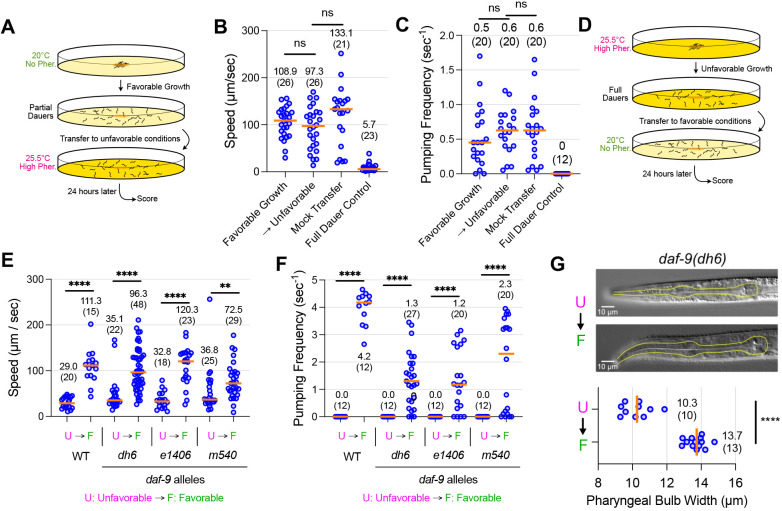
***daf-9* partial dauers may be partially exited dauers.** (A) Experimental schematic to test whether *daf-9(dh6)* partial dauers become full dauers upon transfer to unfavorable conditions. (B,C) *daf-9(dh6)* partial dauers obtained under favorable growth conditions were transferred to unfavorable conditions or again to favorable conditions (mock transfer) and scored 24 h later for speed (B) and pumping frequency (C). Also shown are *daf-9(dh6)* full dauers grown under unfavorable conditions (Full Dauer Control). (D) Experimental schematic to test whether *daf-9(dh6)* full dauers become partial dauers upon transfer to favorable growth conditions. (E,F) *daf-9(dh6)* full dauers were obtained under unfavorable, dauer-inducing growth conditions, transferred to favorable conditions, and then scored 24 h later for speed (E) and pumping frequency (F). *daf-9* alleles *e1406* (null) and *m540* (weak loss-of-function) show similar partial dauer exit phenotypes. (G) *daf-9(dh6)* partially exited dauers obtained via the method in D have wider pharynxes than their full dauer counterparts. Yellow outlines indicate pharynxes. ns, not significant. **P<0.01, ****P<0.0001 (Mann–Whitney test). Horizontal bars and in-graph numbers show median values. Numbers in parentheses indicate number of animals. Each dot is one animal.

To assess the role of steroid hormones in the *C. elegans* dauer exit developmental decision (Fig. 1), we chose to focus on *daf-9*, as it is the only gene acting in a steroid hormone pathway for which null mutants such as *daf-9(dh6)* and *daf-9(e1406)* show completely penetrant Daf-c phenotypes, suggesting that its loss severely abrogates steroid hormone signaling (Antebi, 2015; Gerisch and Antebi, 2004; Gerisch et al., 2001; Rottiers et al., 2006). We first confirmed that *daf-9* null mutants could form full dauers that would be suitable for subsequent dauer exit analysis. Previous reports indicate that the Daf-c steroid hormone mutants *daf-9(dh6)* and *daf-12(rh273)* form full dauers under unfavorable growth conditions (Antebi et al., 1998; Gerisch et al., 2001). To confirm these findings, we grew *daf-9(dh6)* animals under unfavorable conditions, which involves high temperature (25.5°C) and the presence of dauer-inducing pheromone extract (see Materials and Methods). These unfavorable growth conditions yielded *daf-9(dh6)* dauer larvae that matched the characteristics of full dauers formed by wild-type animals: both *daf-9(dh6)* and *daf-9(e1406)* dauers showed no pumping, low motility and a darkened, radially constricted body [Fig. 2 and Movie 1 for *daf-9(dh6)*; Fig. 3E,F for *daf-9(e1406)*]. *daf-9(dh6)* full dauers are also completely SDS resistant – a hallmark of the dauer state (Fig. S1A). In contrast, when *daf-9(dh6)* mutants were grown under favorable conditions, we observed partial dauers that pumped more frequently, moved faster and showed enlarged pharynxes compared with full dauers (Fig. 2). Comparisons with L3 larvae show that these partial dauers have pharynx sizes and pharyngeal pumping rates between those of full dauers and L3 larvae, but their movement speeds are comparable with or faster than those of L3 larvae (Fig. 2).

### Evaluating the *daf-9* partial dauer state

Having confirmed that *daf-9* mutants form partial or full dauers depending on the environmental conditions, we further probed the robust *daf-9* partial dauer phenotype, reasoning that it would provide insights into understanding how steroid hormones regulate the dauer process. We reasoned two likely possibilities for how *daf-9* partial dauers fit into the developmental pathway: (1) Partial dauers are *en route* to becoming full dauers but require unfavorable environmental stimuli to complete the dauer entry process; (2) partial dauers are partially exited dauers that have gone through a full dauer state and then initiate, but cannot complete, dauer exit (Fig. 1).

To test possibility (1), in which *daf-9* partial dauers require unfavorable conditions to become full dauers, we grew *daf-9(dh6)* mutants under favorable conditions to first form partial dauers and then transferred them to unfavorable conditions to determine whether they could form WT-dauers (Fig. 3A-C). We found that, despite a 24-h incubation under unfavorable conditions, *daf-9(dh6)* partial dauers did not transition towards a full dauer state. These *daf-9(dh6)* animals continued to move and pump at high rates in comparison with *daf-9(dh6)* full dauers. Thus, we found it unlikely that the partial dauer state obtained under favorable growth conditions represents a transition state that is *en route* to becoming full dauer.

### *daf-9* partial dauers are likely partially exited dauers

To assess possibility (2), in which *daf-9* partial dauers are first full dauers that then partially exit dauer, we grew *daf-9(dh6)* mutants under unfavorable conditions to first form full dauers, and then we transferred them to favorable conditions to examine whether they became partial dauers. We found that 24 h post-transfer, *daf-9(dh6)* larvae actively pumped, moved significantly more and had wider pharynxes compared with before the transfer (Fig. 3D-G and Movie 2), thereby recapitulating the *daf-9* partial dauer state. These partially exited dauers slowly continued to grow radially and develop a larger pharynx even past the 24-h mark, although they never developed into healthy reproductive adults (Gerisch et al., 2001). We obtained similar results using *daf-9* alleles *e1406* (another putative null mutation) and *m540* (a weaker loss-of-function allele) (Fig. 3E,F). Together, these findings suggest that transfer to favorable conditions causes full *daf-9* dauers to initiate dauer exit and engage in concomitant behavioral and morphological changes, such as increased pumping, motility and pharyngeal expansion.

We also examined whether this partial dauer exit phenotype could be recapitulated at the level of the nuclear hormone receptor DAF-12/FXR. Daf-c alleles of *daf-12* that bear mutations altering the DAF-12 putative ligand binding domain form partial dauers with low penetrance under favorable growth conditions (Antebi et al., 1998, 2000). We analyzed a Daf-c mutant, *daf-12(rh273)*, and found that we were able to induce full dauers under unfavorable growth conditions that could become partial dauers upon transfer to favorable conditions (Fig. S1B,C). Therefore, *daf-12(rh273)* mutants can phenocopy the partial dauer exit phenotype of *daf-9* putative null mutants, consistent with DAF-12/FXR mediating this phenotype.

A feature of dauer exit is its irreversibility: wild-type dauers that have been shifted to favorable conditions commit to dauer exit within 1 h, as shifting them back onto unfavorable conditions afterwards cannot maintain or restore the dauer state (Golden and Riddle, 1984). We asked whether *daf-9(dh6)* partial dauers were in an irreversible state of partial dauer exit, or whether a return to unfavorable conditions could cause the animal to become a full dauer again. We grew *daf-9(dh6)* mutants under unfavorable conditions to induce full dauers, transferred the resulting dauers to favorable conditions to stimulate partial dauer formation, and then transferred them back onto unfavorable conditions to see if they could become full dauers again (Fig. S1D-F). Transfer into unfavorable conditions neither dramatically altered pumping rate nor movement speed compared with the mock transfer control, nor did it produce larvae that were similar to full dauers, even 24 h after a return to unfavorable conditions. This observation suggests that partial dauers may be animals that have committed to, but can only partially complete, dauer exit.

Having concluded that *daf-9(dh6)* partial dauers resemble partially exited dauers, we compared the temporal progression of dauer exit in wild-type versus *daf-9(dh6)* animals (Fig. S2). When wild-type and *daf-9(dh6)* dauers are transferred to favorable conditions, they both gradually develop dauer exit behaviors and morphologies such as increased pumping, movement speed and pharyngeal expansion. *daf-9(dh6)* dauers develop these exit characteristics more slowly than do wild-type dauers. With the exception of movement speed, *daf-9(dh6)* partial dauers at 24 h post-transfer resemble wild-type partially exited dauers at over 8 h post-transfer (Fig. S2). This delay suggests that *daf-9* may be involved in the pace with which these exit characteristics manifest. As for movement speed, *daf-9(dh6)* partially exited dauers move at higher speeds than wild-type dauers do after 8 h following transfer to favorable conditions. These data highlight the similarities between *daf-9(dh6)* partial dauers and wild-type animals during the early stages of dauer exit.

### Assessing whether *daf-9* partial dauers pass through a transient state of full dauer

**Fig. 4.**
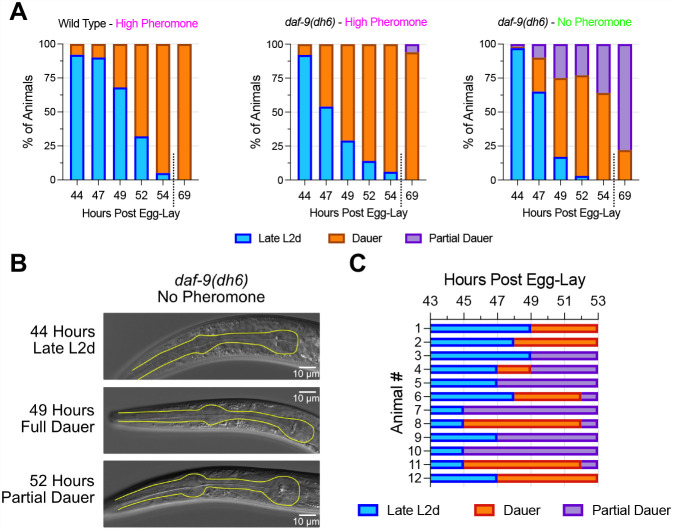
***daf-9(dh6)* larvae transiently become full dauers in the absence of exogenous pheromone.** (A) *daf-9(dh6)* worms were grown in the presence or absence of exogenously added pheromone and then scored as late L2d, full dauer or partially exiting dauer animals. *n*≥53 animals for all observations. (B) Representative images of pharynxes from worms grown as in A. Note the shrinkage of the posterior pharyngeal bulb at 49 h, indicative of full dauer status, followed by an enlargement of the pharynx at 52 h, indicative of partial dauer exit. Yellow outlines indicate pharynxes. (C) *daf-9(dh6)* worms grown as in A, but worms were individually grown and observed every 2 h starting at 43 h post egg-lay. Seven out of 12 animals could be observed in a full dauer state.

Under the hypothesis that *daf-9* partial dauers were once full dauers that then partially exited, it should be possible to observe *daf-9* mutants pass through a period of being full dauers before they become partial dauers even under favorable conditions. We grew *daf-9(dh6)* mutants under favorable conditions and scored animals every 2 h as being late L2d, full dauer or partial dauer based on metrics such as pharyngeal pumping, locomotion and morphology (Fig. 4A,B, and see Materials and Methods). As controls, we also grew *daf-9(dh6)* and wild-type animals under unfavorable conditions in parallel. To maintain synchrony across the different growth conditions, we grew all animals at a high temperature of 25.5°C but withheld pheromone from the *daf-9(dh6)* mutants grown under favorable conditions. Although growth at 25.5°C favors dauer formation, it alone cannot induce dauer formation in wild-type animals (Ailion and Thomas, 2000). At 44 h post egg-lay, the vast majority of animals were late L2d (Fig. 4A). By 49 h, ∼50% of *daf-9(dh6)* mutants grown in the absence of pheromone could be scored as full dauers, whereas by 52 h, 75% of animals were found to be full dauers. By 69 h, the majority of animals were partial dauers. In contrast, both the wild-type and *daf-9(dh6)* animals grown under high pheromone conditions showed a steady increase in the proportion of full dauers over time and few, if any, partial dauers could be found at any time point (Fig. 4A). These results show that a proportion of *daf-9(dh6)* mutants grown in the absence of pheromone become full dauers for some period of time.

To determine what fraction of *daf-9(dh6)* mutants pass through a transient dauer state, we repeated the above experiment but with single animals. We grew *daf-9(dh6)* mutants without pheromone at 25.5°C and, after 43 h post egg-lay, we transferred the resulting late L2ds onto new plates without pheromone (one per plate) and we scored individual animals over time (Fig. 4C). In concordance with our bulk tracking assay, we observed full dauers between 45 and 50 h that later became partial dauers. Of 12 tracked animals, we observed seven that went through a period of being full dauers. For these animals, we observed an L2d molt in which the animal detached from and sometimes rolled inside its cuticle (Singh and Sulston, 1978). Afterwards, the animal would cease both movement and pharyngeal pumping before completing radial constriction to become a full dauer. Within a few hours, these dauers slowly began pumping and moving more (a sign of partial dauer exit), but radial expansion did not occur until many hours later. Some animals were never observed as having formed full dauers (Fig. 4C), which may be because their transition through full dauers occurred in between time points or because they skipped the full dauer state.

We also performed the above single animal observation experiments under more favorable conditions by lowering the temperature to 20°C. However, under these conditions we were unable to find any *daf-9(dh6)* larvae that went through a full dauer state, despite making observations every hour (Fig. S3). *daf-9(dh6)* mutants grown under these conditions passed through an L2d stage and L2d molt indistinguishable from that of wild-type L2d larvae and L2d larvae formed by *daf-9(dh6)* mutants grown under unfavorable conditions. Following the L2d molt, these *daf-9(dh6)* mutants instead passed through an intermediate state that involved both elements of being a dauer (a darkened body) as well as a partial dauer (pumping, motility), before becoming well-recognizable partial dauers usually within 1 h. These observations suggest that high temperatures facilitate formation of full dauers in *daf-9(dh6)* mutant animals in the absence of exogenously added pheromone.

### Genetic and physiological factors that could affect partial dauer formation

**Fig. 5.**
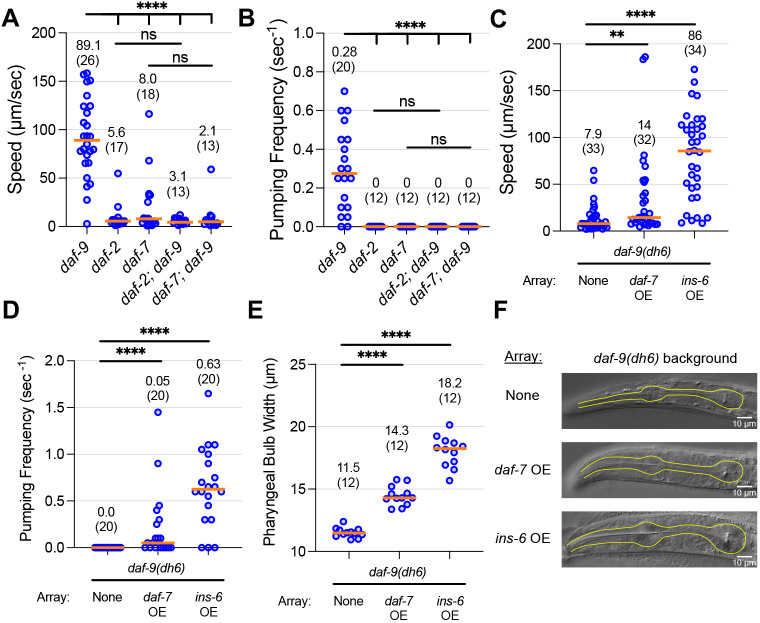
**Effects of *daf-2* and *daf-7* on the *daf-9* partial dauer phenotype.** (A,B) Single and double mutants between *daf-9(dh6)* and *daf-2(e1370)* or *daf-7(e1372)* were grown under favorable conditions. Although *daf-9(dh6)* worms form partial dauers, *daf-2(e1370)*, *daf-7(e1372)* and their respective *daf-9(dh6)* double mutants do not form partial dauers. ****P<0.0001 (Mann–Whitney test between *daf-9* single mutant and all other double mutants). (C-F) Overexpression of *daf-7* or *ins-6* in a *daf-9(dh6)* background is sufficient for partial dauer phenotypes. *daf-9(dh6)* mutants with or without an extrachromosomal array that pan-neuronally overexpresses (OE) *daf-7* or *ins-6* were grown under unfavorable conditions. Although animals without the array form full dauers, animals overexpressing *daf-7* or *ins-6* exhibit weaker or stronger partial dauer phenotypes, respectively. Phenotypes measured include speed (C), pumping frequency (D) and terminal pharyngeal bulb width (E,F). Yellow outlines indicate pharynxes. ns, not significant. **P<0.01, ****P<0.0001 (Mann–Whitney test). Horizontal bars and in-graph numbers show median values. Numbers in parentheses indicate number of animals. Each dot is one animal.

We sought to characterize the genetic and physiological underpinnings of the *daf-9* partial dauer exit state. We asked whether the *daf-9(dh6)* partial dauer exit phenotype was dependent on other genes in the dauer pathway by performing double mutant analysis of *daf-9(dh6)* with strong loss-of-function mutations in the insulin pathway gene *daf-2(e1370)* (encoding a homolog of the insulin growth factor receptor) and the TGF-β pathway gene *daf-7(e1372)* (encoding an ortholog of human GDF11). Mutants in *daf-2* and *daf-7* possess strong Daf-c phenotypes and form full dauers in the absence of exogenous pheromone at high temperatures. We grew *daf-2(e1370); daf-9(dh6)* and *daf-7(e1372); daf-9(dh6)* double mutants under favorable conditions alongside *daf-9(dh6)*, *daf-2(e1370)* and *daf-7(e1372)* single mutants to see which would form partial dauers (Fig. 5A,B). We found that only *daf-9(dh6)* formed partial dauers under these conditions, whereas the *daf-2(e1370); daf-9(dh6)* and *daf-7(e1372); daf-9(dh6)* double mutants were phenotypically identical to the *daf-2(e1370)* and *daf-7(e1372)* single mutants in that they formed full dauers. These results indicate that the *daf-9(dh6)* partial dauer phenotype is dependent on the insulin pathway as well as the TGF-β pathway.

We also evaluated whether stimulation of the insulin and TGF-β pathways was sufficient to induce partial dauer phenotypes. To do so, we overexpressed *ins-6* and *daf-7* pan-neuronally in a *daf-9(dh6)* background. *ins-6* encodes an insulin-like peptide shown to activate the insulin pathway and promote dauer exit (Cornils et al., 2011; Hua et al., 2003). We grew these transgenic animals under unfavorable conditions to induce full dauer development, and we found that animals overexpressing *daf-7* or *ins-6* exhibited partial dauer phenotypes (Fig. 5C-F). Overexpression of *daf-7* weakly increased locomotion speed, pumping frequency and pharyngeal expansion, whereas overexpression of *ins-6* strongly bolstered these traits. These phenotypes were not due to abnormal growth or dauer development defects as these transgenic animals underwent a wild-type L2d molting process before becoming partial dauers. Taken together, these data suggest that the insulin and TGF-β pathways are necessary and sufficient for partial dauer formation in a *daf-9(dh6)* background.

We speculated that a potential reason for the partial dauer exit phenotype could be that a small amount of reproduction-promoting steroid hormones continues to be produced even in *daf-9* putative null mutants, and that these steroid hormones might trigger partial dauer exit. We reasoned that withholding cholesterol, a precursor for the vast majority of DAF-12 steroid hormone ligands (Aguilaniu et al., 2016), could hinder partial dauer exit. We found that withholding cholesterol from the NGM media did not hinder the formation of partial dauers (Fig. S4), suggesting that the partial dauer state is not a result of residual steroid hormone production. We cannot rule out the possibility that there was sufficient cholesterol or sterol derivatives contained in the medium and/or passed on by previous generations to induce a partial dauer exit state.

### *daf-9* dependent steroid hormone biosynthesis is necessary for and promotes dauer exit

**Fig. 6.**
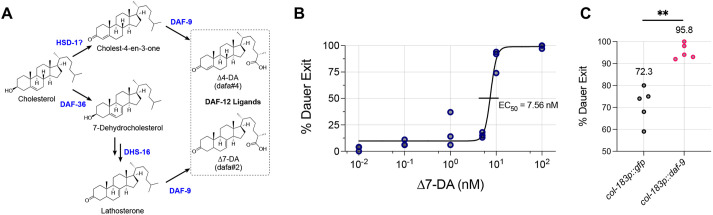
**The *daf-9* steroid hormone pathway is necessary for and promotes dauer exit.** (A) Steroid hormone biosynthetic pathway. DAF-9 catalyzes the final oxidation step required to form all known endogenous ligands of DAF-12, which promote reproductive development over dauer. Figure adapted from Mahanti et al. (2014) and Aguilaniu et al. (2016). (B) Dose response curve for the efficacy of Δ7-DA to rescue the dauer exit defect of *daf-9(dh6)* mutants. *daf-9(dh6)* full dauers were transferred to favorable conditions containing various concentrations of Δ7-DA, and 24 h later animals were scored for complete dauer exit. Lower concentrations yielded partially exited dauers, whereas 100 nM induced nearly all animals to completely exit dauer. (C) Overexpression of *daf-9* from the hypoderm-specific and dauer-specific promoter *col-183p* in a wild-type background promotes dauer exit. For B and C, each dot is a biological replicate of scored dauer exit plates, with each group having a total *n*≥100 animals. **P<0.01 (bootstrapped permutation test using 10^4^ samples).

Having confirmed that steroid hormone mutants retain the ability to form full dauers, we proceeded to assess the role of the steroid hormone biosynthesis pathway in dauer exit using *daf-9(dh6)* full dauers. *daf-9* encodes a cytochrome P450 enzyme that catalyzes the formation of all known steroid hormones (Fig. 6A) (Motola et al., 2006). Among the DA, Δ7-DA has been shown to rescue the Daf-c phenotype of *daf-9(dh6)* mutants by allowing them to bypass dauer entry to become healthy adults (Mahanti et al., 2014; Motola et al., 2006). We determined whether Δ7-DA could also rescue the partial dauer exit phenotype of *daf-9(dh6)* mutants (Fig. 6B). We let *daf-9(dh6)* full dauers form under unfavorable growth conditions and then transferred them to favorable conditions with varying concentrations of Δ7-DA and scored for complete dauer exit the next day. At low Δ7-DA concentrations, animals become partial dauers, whereas at 100 nM Δ7-DA nearly all *daf-9(dh6)* mutants fully exit dauer and resume reproductive development. Nonlinear regression analysis of the dose response curve reveals an EC_50_ of 7.56 nM. We also found that we could induce complete dauer exit in *daf-9(dh6)* partial dauers, obtained by exposing full dauers to favorable conditions for 24 h, via incubation with 100 nM Δ7-DA (78% become gravid adults within 2 days, *n*=346).

We also determined whether Δ7-DA could induce dauer exit of *daf-9(dh6)* mutants in the presence of pheromone. Even at 100 nM Δ7-DA, almost all animals remained full dauers (89.4%, *n*=284). This could be because Δ7-DA is insufficient to induce dauer exit without the dauer first being exposed to favorable conditions that activate insulin and TGF-β pathways. Another possibility could be that their lack of feeding and/or their thickened cuticle (Cassada and Russell, 1975) preclude access to Δ7-DA.

We examined whether overexpression of *daf-9* was sufficient to induce dauer exit in a wild-type background. As constitutive and ubiquitous overexpression of *daf-9* would likely preclude dauer formation, we drove expression of *daf-9* cDNA from the *col-183* promoter (Shih et al., 2019). *col-183* shows maximal expression during dauer and within the hypodermis, a tissue that exhibits high *daf-9* expression levels during reproductive development (Gerisch et al., 2001; Schaedel et al., 2012). Overexpression of *daf-9* from the *col-183* promoter significantly increased the fraction of dauers that exited, indicating that hypodermal *daf-9* expression during dauer promotes dauer exit (Fig. 6C).

### Spatiotemporal regulation of *daf-9* during dauer exit resembles that of dauer entry

**Fig. 7.**
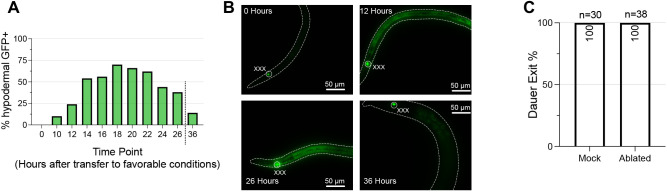
**Spatiotemporal dynamics of *daf-9* expression during dauer exit.** (A) Time course of hypodermal *daf-9::gfp* expression during dauer exit. Dauers expressing a *daf-9::gfp* translational fusion were transferred to favorable conditions and imaged for the presence or absence of GFP signal in the hypoderm. *daf-9::gfp* is expressed in the highest percentage of animals at ∼16-20 h after transfer to favorable conditions. *n*≥20 for all time points. (B) Representative images of animals observed in A, with bodies outlined and the worm bodies neuroendocrine cells (which constitutively express *daf-9::gfp*) labeled. Note the increase in DAF-9::GFP in images for 12 and 26 h. Dashed lines outline worm bodies. (C) Laser ablation of XXX cells does not prevent dauer exit. Following ablation of the XXX cells using a laser microbeam, dauers and mock-ablated control dauers were transferred to favorable conditions and scored for dauer exit 24 h later.

We characterized the spatiotemporal regulation of *daf-9* during dauer exit to examine whether it differs from that during dauer entry. Before the dauer entry decision, *daf-9* is expressed exclusively in the XXX neuroendocrine cells. When animals decide to enter the reproductive life cycle, *daf-9* expression increases throughout the hypodermis (Gerisch et al., 2001; Schaedel et al., 2012). To test whether this expression pattern holds true during dauer exit, we used the same DAF-9::GFP translational fusion-bearing strain (Gerisch et al., 2001) and monitored GFP expression as animals exited dauer (Fig. 7A,B). Upon shifting dauer larvae from unfavorable to favorable conditions to induce exit, we observed an increase in the proportion of animals displaying hypodermal GFP expression. The proportion peaked at 18 h post-shift, which is when larvae have nearly entered L4, at which point nearly 75% of animals showed hypodermal GFP. After 36 h post-shift, the vast majority of animals (∼90%) lost all hypodermal GFP but retained GFP expression in the XXX cells. Thus, the spatiotemporal dynamics of *daf-9* expression for the dauer exit decision seem to match those of the dauer entry decision in that there is widespread hypodermal *daf-9* expression as the animal chooses the reproductive route during development.

The constitutive expression of *daf-9* in the XXX cells throughout dauer led us to ask whether the XXX cells might be important for dauer exit. Published reports indicate that if the XXX cells are ablated by a laser microbeam during the L1 stage, only a small fraction of animals become partial dauers, even under favorable conditions (Gerisch et al., 2001; Ohkura et al., 2003). However, if ablation occurs during the L2d stage after the animals have been reared under unfavorable conditions, then nearly all larvae form dauers even after a shift to favorable conditions (Schaedel et al., 2012). To examine whether the XXX cells are dispensable for dauer exit, we bilaterally ablated the XXX cells in dauers using a laser microbeam in dauer animals and transferred the ablated animals to a recovery plate under favorable conditions to induce dauer exit. We found that all XXX-ablated dauer larvae were able to exit dauer, similar to their mock ablated counterparts (Fig. 7C). To validate this finding, we also genetically ablated the XXX cells by expressing the human caspase gene *ICE* from the XXX-specific promoter *eak-3p* using the cGAL bipartite expression system for *C. elegans* (Wang et al., 2017). While we observed some artifacts owing to the *UAS::ICE* transgene, such as formation of SDS-sensitive dauers that exited dauer at rates higher than the cGAL XXX cell-specific driver strain, genetic ablation of the XXX cells did not substantially prevent dauer larvae from exiting more when compared with control animals expressing the *UAS::ICE* effector transgene without the XXX cell-specific driver (Fig. S5). These findings suggest that the XXX cells may not be essential for dauer exit.

## DISCUSSION

### Partial dauers formed by steroid hormone mutants are likely partially exited dauers

We evaluated how the steroid hormone pathway regulates both the dauer entry and dauer exit developmental decisions by first addressing why steroid hormone mutants such as *daf-9* form partial dauers. Our evidence favors the hypothesis that these partial dauers are dauers that have commenced but cannot complete dauer exit. We find that forming *daf-9* full dauers under unfavorable conditions followed by transfer to favorable conditions to induce dauer exit produces animals that resemble the partial dauers that are formed when *daf-9* mutants are grown constantly under favorable conditions (Figs 2 and 3E-G). We also show that even when pheromone is omitted from the growth medium, some *daf-9(dh6)* animals pass through a state of full dauer before initiating dauer exit owing to the favorable environment (Fig. 4). But given the lack of reproduction-promoting steroid hormones such as Δ7-DA, these larvae can only partially exit from dauer, resulting in a partial dauer state that slowly grows to unhealthy and sterile adulthood.

Under completely favorable conditions (i.e. no pheromone and low temperature), *daf-9(dh6)* larvae could not be found in a full dauer state (Fig. S3). Following the L2d molt, we were only able to find *daf-9(dh6)* mutants in a transient, intermediate state that looked like a hybrid between an L2d and a partial dauer in terms of morphology and behavior. Within 1 h, these animals then quickly went on to become familiar partial dauers. We could not observe a similar intermediate state in wild-type animals, which we attempted to do by transferring wild-type L2d larvae that had committed to becoming dauers from unfavorable to favorable conditions (Schaedel et al., 2012). Instead, these animals passed through a full dauer state. These observations suggest that *daf-9(dh6)* mutants skip or fail to enter the full dauer state under favorable conditions. One possibility for this observation is that constant growth under favorable conditions activates insulin and TGF-β pathways in *daf-9(dh6)* mutants and prevents full dauer formation. Alternatively, *daf-9* may be required for full dauer formation under favorable conditions but not unfavorable conditions. Untangling these possibilities requires a better understanding of the molecular effectors downstream of the insulin, TGF-β and steroid hormone pathways that are directly responsible for the behavioral and morphological changes associated with full dauer formation.

### The *daf-9* partial dauer state provides new insights into the dauer exit process

Recognizing that the *daf-9* partial dauer state may be a partially exited dauer prompts a consideration of what genetic, developmental and physiological factors may be responsible for partial dauer exit. We considered that residual steroid hormone production, either through non-*daf-9*-dependent biosynthesis or transgenerational rescue, suffices to trigger partial dauer exit. The former is improbable because there are no characterized biochemical pathways to form DAF-12 ligands that do not involve DAF-9 (Aguilaniu et al., 2016). The latter appears to be unlikely because growing *daf-9(dh6)* mutants on media lacking cholesterol does not suppress partial dauer formation (Fig. S4). Moreover, a double mutant defective in both *daf-9* and *daf-36*, which would presumably have lower steroid hormone levels than *daf-9* single mutants alone, phenocopies *daf-9* to produce partial dauers (Rottiers et al., 2006).

The partial dauer exit phenotype can be suppressed by mutations in important components of the insulin or TGF-β pathways, and a partial dauer state can be induced by activation of either of these two pathways (Fig. 5). This is consistent with a model in which steroid hormone mutants form partial dauers because insulin and TGF-β pathways are activated in response to the animal sensing favorable conditions, subsequently triggering initial dauer exit behaviors and morphologies. This model is further supported by the fact that the transcriptional outputs of the insulin pathway, mediated by DAF-16/FOXO, are distinct from those of DAF-12/FXR, although the two pathways have significant crosstalk (Jeong et al., 2010). Whereas DAF-16/FOXO directly regulates the transcription of genes related to metabolism, stress resistance and longevity, DAF-12/FXR is known to transcriptionally regulate heterochronic gene pathways that govern developmental timing (Hochbaum et al., 2011; Kumar et al., 2011). Given these different transcriptional networks, one hypothesis is that favorable conditions activate insulin and TGF-β pathways that transcriptionally upregulate target genes to initiate dauer exit. However, without liganded DAF-12/FXR activity to stimulate heterochronic gene expression, the larvae cannot proceed to reproductive adulthood and remain as partial dauers. Supplementation of Δ7-DA to these partial dauers rescues this exit defect and promotes complete dauer exit.

In summary, our results are consistent with a model in which dauer exit comprises two stages. The first stage involves the transition from a full dauer to a partially exited dauer and is not dependent on *daf-9* but is instead mediated by insulin and TGF-β signaling (Fig. 5). As insulin and TGF-β pathway ligand-encoding genes are regulated in response to dauer-specific cues such as pheromone and food levels (Li et al., 2003; Ren et al., 1996), this first stage could be considered a ‘sensory integration’ step in the dauer exit decision. The second stage in this dauer exit model describes the transition from a partially exited dauer to a reproductive L4 larvae and is mediated by the steroid hormone pathway (Fig. 6). Because this stage encompasses the important developmental steps that entail escape from diapause into reproduction, it could be considered the ‘execution’ step in the dauer exit decision. Further experiments that manipulate insulin, TGF-β and steroid hormone pathway activity in full and partial dauers with temporal precision will help evaluate such a model.

It remains an open question as to whether other described partial dauers, such as those produced by double mutant strains carrying *daf-16* (Ailion and Thomas, 2000; Vowels and Thomas, 1992), are identical to the partial dauers formed by steroid hormone mutants. One distinction is that *daf-16* double mutant partial dauers were described to quickly and spontaneously exit to adulthood (Vowels and Thomas, 1992), whereas *daf-9(dh6)* partial dauers cannot ever fully exit. We have not rigorously tested those strains using our partial dauer exit analyses.

### Steroid hormone biosynthesis governs dauer exit in a manner similar to the L1 to L2 versus L2d dauer entry subdecision

We evaluated how the *C. elegans* steroid hormone pathway regulates dauer exit in comparison with dauer entry. We established that steroid hormones are essential for full dauer exit by showing that *daf-9(dh6)* dauers only partially exit in the absence of Δ7-DA but completely exit when supplemented with Δ7-DA at nanomolar concentrations (Fig. 6B). Previous dose response curves showing the relationship between Δ7-DA and the Daf-c dauer entry phenotype of *daf-9(dh6)* animals show an EC50 of ∼5-25 nM, whereas 100 nM fully rescues the dauer entry phenotype (Schaedel et al., 2012). These results suggest that similar concentrations of Δ7-DA mediate both dauer entry and dauer exit in *daf-9(dh6)* animals. We further show that hypodermal overexpression of *daf-9* during dauer promotes dauer exit (Fig. 6C), therefore demonstrating a parallel role for *daf-9-*dependent steroid hormones in regulating both dauer entry and dauer exit.

In comparing how the steroid hormone pathway regulates dauer exit versus dauer entry, our results suggest that the role of the steroid hormone pathway in dauer exit closely mirrors its role in the L1 to L2 versus L2d dauer entry subdecision (see Fig. 1A). First, our *daf-9::gfp* translational fusion analysis shows that hypodermal upregulation of *daf-9::gfp* begins at ∼10-14 h following transfer of dauers onto favorable conditions (Fig. 7A). This delay in hypodermal *daf-9::gfp* expression nearly matches that of the L1 to L2 versus L2d decision, in which it was shown that *daf-9::gfp* expression increased starting at 24-27 h post hatch in animals grown under favorable conditions, whereas larvae commit to reproductive adulthood much earlier at ∼14-16 h post hatch (Schaedel et al., 2012). In stark contrast, during the L2d to dauer versus L3 decision, hypodermal *daf-9::gfp* expression closely aligned with the time window in which L2d larvae committed to reproductive adulthood (Schaedel et al., 2012). Importantly, given that dauer exit commitment occurs within 1-2 h following transfer onto favorable conditions (Golden and Riddle, 1984), the fact that hypodermal DAF-9::GFP fluorescence does not appear until hours later suggests that hypodermal upregulation of *daf-9* may be a consequence of, rather than a cause of, the commitment to exit dauer.

Second, our XXX ablation experiments argue against an essential role for the XXX cells in dauer exit, as ablation of XXX cells does not prevent dauers from exiting (Fig. 7C and Fig. S5). Such observations are consistent with the nonessential role of XXX cells in the L1 to L2 versus L2d decision, in which groups have reported that ablation of XXX cells in L1 larvae has only a minor effect on dauer entry (Gerisch et al., 2001; Ohkura et al., 2003). In contrast, the XXX cells are required for reproductive adulthood in the L2d to L3 versus dauer subdecision, as ablation of the XXX cells in L2d larvae prevents development into the L3 stage even under favorable conditions (Schaedel et al., 2012).

### Hormonal regulation of diapause entry and exit in other organisms

The DAF-12 steroid hormone pathway in *C. elegans* is conserved in other parasitic nematode species, the infective larvae stage of which are comparable with *C. elegans* dauers. Treatment of multiple parasitic species during early larval growth with Δ7-DA prevents entry into the infective stage, whereas treatment of infective larvae prompts exit from the infective stage (Ogawa et al., 2009; Wang et al., 2009), mirroring our results using Δ7-DA to induce full dauer exit (Fig. 6B). Such conservation suggests that mechanistic knowledge of how steroid hormones control dauer exit in *C. elegans* could yield potential therapeutic insights to combat other parasitic species.

Diapause is evolutionarily conserved and phylogenetically widespread, and hormonal control of both diapause entry and exit is especially well-studied in insects (Denlinger et al., 2012). In *Heliothis* and *Helicoverpa* species of moth, diapause entry is likely caused by insufficient levels of diapause hormone (DH) and prothoracicotropic hormone (Xu and Denlinger, 2003). Administration of DH or DH mimics to diapausing pupae results in diapause termination, indicating that DH is sufficient to cause diapause exit (Zhang et al., 2011). The notion that diapause occurs in the absence of a pro-development hormone(s), and terminates in its presence, may therefore be conserved between nematodes and insects, but in some insect species in which diapause is maternally controlled, diapause entry and exit are regulated via different hormonal processes. Studies of embryonic diapause in the silkworm *Bombyx mori* have demonstrated a central role for DH in triggering, rather than preventing, diapause in developing embryos (Sato et al., 1992). Diapause termination, on the other hand, does not appear to depend on the absence of DH but instead on the presence of pro-development ecdysteroids such as 20-hydroxyecdysone (Iwata et al., 2005; Sonobe and Yamada, 2004). In *C. elegans* and other animal species, co-opting the same hormonal signaling process for preventing diapause entry and inducing diapause exit could be an adaptive strategy that efficiently uses pre-existing molecular pathways for multiple purposes.

## MATERIALS AND METHODS

### *C. elegans* strains and maintenance

*C. elegans* strains were derived from the wild-type strain N2 (Bristol) and were cultured according to standard laboratory conditions on Nematode Growth Medium (NGM) agar seeded with *Escherichia coli* OP50 as the food source. A list of strains used in this study, including their genotypes and origins, can be found in Table S1. Maintenance and propagation of *C. elegans*, with the exception of *daf-9* loss-of-function mutants, were performed under typical growth conditions with NGM agar seeded with *E. coli* OP50 cultures as described previously (Brenner, 1974). The *daf-9(dh6)*, *daf-9(e1406)* and *daf-9(m540)* mutants were propagated in the presence of 100 nM Δ7-DA to promote reproductive development. The *daf-9(dh6)* strain was obtained by propagating non-array carrying animals from PS5511 {*daf-9(dh6); dhEx24[T13C5, pTG96(+)]*} on Δ7-DA.

### Dauer entry induction

To induce full dauers in wild-type and *daf-9* mutants, 10-20 young adults were placed on 35 mm diameter Petri dishes containing 2 ml of NGM agar (without peptone) supplemented with a quantity of crude pheromone extract (Schroeder and Flatt, 2014) that normally induces 95-100% of dauers in wild-type animals – typically 10-30 µl per 2 ml of agar. Plates were seeded with 10 µl of 8% w/v *E. coli* OP50 that was heat-killed at 95°C for 5 min. Adults were picked onto the plate and allowed to lay eggs at room temperature (RT; 22-23°C) for 5-9 h before being removed, during which time they typically laid 200-300 eggs. The plates were then further seeded with an additional 20 µl of heat-killed OP50. Afterwards, the plates were wrapped with parafilm and incubated at 25.5°C for 60-72 h.

### Dauer exit assay

Dauers were formed according to ‘dauer entry induction’, above. In most cases, dauers were selected for by an SDS wash (2%, 30 min, 25°C) to kill non-dauers before being washed 3× in M9 solution (1 min, RT, 1000 ***g***). Surviving dauers were then plated onto dauer exit plates, which were identical to dauer entry plates but contained a lower concentration of crude pheromone extract (typically 0.5-2 µl per 2 ml of agar) that had been determined to induce ∼40-60% of wild-type dauers to exit within 24 h. In the case of the genetic ablation assay using the *UAS::ICE* construct, the SDS wash step was omitted as dauers bearing the *UAS::ICE* construct were SDS sensitive. Dauers were instead washed directly onto the dauer exit plate. Following 24 h after dauers were transferred onto dauer exit plates, larvae were scored as having exited dauer if they showed any pharyngeal pumping or if their body had thickened and lightened considerably. Additional factors that favored scoring an animal as having exited dauer included whether the larva showed foraging behavior (such as increased head turns) or increased and consistent locomotory behavior, both of which are absent from dauers.

### Microscopy and image analysis

Worms were immobilized on a 4-10% agarose pad (10% for dauer imaging, 4% for others) in 1-2 µl of 10 mg/ml levamisole, 50 mM sodium azide or 0.1 µm polystyrene beads (Polysciences) for dauer imaging. Imaging was performed on a Zeiss AxioImager2 equipped with a Colibri 7 for LED fluorescence illumination and an Axiocam 506 Mono camera (Carl Zeiss). Pharyngeal bulb width measurements were performed using Zen Blue 2.3 (Zeiss) software using the length tool to measure the widest section of the posterior pharyngeal bulb. Images were processed using FIJI (ImageJ). Pharyngeal outlines were drawn using Affinity Designer (Serif). Differential interference contrast (DIC) microscopy images without the pharyngeal outlines can be found in Fig. S6.

### Laser ablation in dauers

PS8568 animals expressing *gfp* in the XXX cells were induced to form dauers and then immobilized on a 4% agarose pad with 10 mg/ml levamisole. Laser ablation was performed on a Zeiss Axioskop (Carl Zeiss) equipped with an Andor MicroPoint nitrogen-pulsed laser microbeam (Oxford Instruments). XXX cells were visualized under fluorescence and the laser was fired at ∼5 Hz for a total of 20-30 pulses, or until all discernable fluorescence was gone. Cellular damage could often be visualized under DIC. Following surgery, animals were recovered onto a 35 mm NGM plate seeded with OP50 washed in S Basal and scored for dauer exit 24 h later. GFP was no longer discernable under stereomicroscopy in successfully ablated animals. Mock ablated animals were prepared and rescued identically to ablated animals but without receiving laser treatment.

### Partial dauer induction

Partial dauers of *daf-9* mutants were successfully obtained using two methods. The first method (favorable growth) involved placing 10-20 young adults on 35 mm Petri dishes containing 2 ml of NGM agar (without peptone) seeded with 10 µl of 8% w/v OP50, washing twice in S Basal and including 50 µg/ml streptomycin to limit bacterial growth (Golden and Riddle, 1984). Adults were allowed to lay eggs at RT before being removed, after which an additional 20 µl of 8% S Basal-washed OP50 was added. Plates were wrapped in parafilm and grown at 20°C for 60-72 h to yield partial dauers. The second method (unfavorable growth followed by transfer to favorable conditions) involved forming dauers according to ‘dauer entry induction’ above. Dauers were then washed in 2% SDS (30 min, 25°C) before being washed 3× in M9, collected by centrifugation (1 min, RT, 1000 ***g***) and then plated onto 35 mm NGM Petri plates lacking pheromone. Partial dauers could be found 24 h later.

### Behavioral scoring

For all behavioral scoring events, animals were allowed to acclimate to RT for 30 min before scoring. For pumping frequency scoring, animals were manually scored under a stereomicroscope at 100× magnification over a 20 s second period. A pumping event was scored as a contraction of the pharyngeal grinder. For locomotion analysis, 1 min videos were recorded and analyzed using the WormLab Imaging System and software (MBF Bioscience). Videos contained on average 8-12 animals per recording event, and multiple videos were pooled together for each experiment. Recordings were performed in areas of the plate that were away from food to maximize contrast, as recordings performed on food prevented accurate tracking of animals. In all cases, the peristaltic speed (absolute) output, measured in µm/sec, was reported for these experiments. We note that the non-zero absolute speed output of many plotted dauers (i.e. Fig. 3) were a result of noise owing to unstable camera movements. These dauers were often perfectly still when viewed by eye (see Movie 1).

### Time course tracking of dauer status in *daf-9(dh6)* mutants

Wild-type and *daf-9(dh6)* full dauer controls were grown according to ‘dauer entry induction’ above. Simultaneously, *daf-9(dh6)* mutants were grown in the absence of exogenous pheromone. All strains were grown at 25.5°C in order to maximize developmental synchrony across the different conditions. For single animal tracking of *daf-9(dh6)* partial dauers, individual L2ds grown under the conditions described above were picked onto new non-pheromone-containing plates starting at 43 h post egg-lay and scored every 2 h. An animal was scored as being a partial dauer if any pumping was observed and/or the body thickened and lightened compared with a normal dauer.

### Cholesterol deprivation

Cholesterol-deprived plates were made similarly to normal dauer plates (i.e. in 35 mm Petri dishes with 2 ml NGM agar without peptone) except that ethanol solvent was added in place of cholesterol. This method was sufficient to enhance the Daf-c phenotype of *daf-9(m540)* mutants, as previously described (Jeong et al., 2010). More severe methods of cholesterol starvation, such as using agarose in place of agar and passaging the animals over two generations in the absence of cholesterol (Gerisch et al., 2001), yielded unhealthy larvae that could not grow to become dauers.

### Double mutant analysis between *daf-9(dh6)* and *daf-2(e1370)* or *daf-7(e1372)*

Double mutants were constructed as follows. Wild-type males were mated to the balancer strain *sC1(s2023) [dpy-1(s2170) umnIs21]*, which are marked by a recessive Dumpy (Dpy) phenotype and a dominant pharyngeal GFP phenotype. The resulting male sC1 heterozygotes were mated to *daf-9(dh6); dhEx24* hermaphrodites. *dhEx24* is an extrachromosomal array containing the cosmid T13C5, containing a rescuing wild-type copy of the *daf-9* locus and a *sur-5p::gfp* marker expressing GFP throughout the body*.* Hemizygous *daf-9(dh6)/*0 male progeny with both whole-body GFP+ (from *dhEx24*) and pharyngeal GFP+ (from *sC1*) were mated again to *daf-9(dh6) dhEx24* hermaphrodites, and the Dpy F_2_ double GFP+ progeny were obtained to yield *sC1(s2023) [dpy-1(s2170) umnIs21]; daf-9(dh6); dhEx24*. This strain was then crossed into a *daf-2(e1370)* or *daf-7(e1372)* background to yield *daf-2(e1370)/sC1(s2023) [dpy-1(s2170) umnIs21]; daf-9(dh6); dhEx24* or *daf-7(e1372)/sC1(s2023) [dpy-1(s2170) umnIs21]; daf-9(dh6); dhEx24*, respectively*.* The *sC1* balancer could then be used to follow *daf-2(e1370)* and *daf-7(e1372)* in trans to facilitate the construction of double mutants between these mutations and *daf-9(dh6)*. For pharyngeal pumping and locomotion assays, the balanced double mutants were grown from eggs at 25.5° without pheromone for 3 days, and *daf-2(e1370); daf-9(dh6)* or *daf-7(e1372); daf-9(dh6)* dauer larvae were identified by looking for non-GFP dauers and picked onto new plates. These dauers were allowed to acclimate on the new plates for at least 30 min before scoring.

### Statistical analysis and plotting

Plots were designed using Prism 9.0 (GraphPad). All plots are representative of at least two independent experiments. Dose response curves for steroid hormones were calculated using the Prism nonlinear regression tool [(Agonist) versus response] with four parameters and the EC50 parameter constrained to be greater than zero. Mann–Whitney tests were performed in Prism. Permutation tests for dauer exit proportion between two samples were performed by first binarizing dauer exit data, pooling the two samples together and simulating experiments by drawing two samples out of the pooled binarized data. The *P*-value was calculated by comparing the number of simulated experiments, out of 10^4^, in which the difference between the simulated proportions was greater than the observed difference between the actual proportions.

## Supplementary Material

Supplementary information

Reviewer comments
