# Supplementary material for: Both entry to and exit from diapause arrest in Caenorhabditis elegans are regulated by a steroid hormone pathway
Source: Development. 2022 May 3;149(9):dev200173. doi: 10.1242/dev.200173 (PMC9148571; doi:10.1242/dev.200173)
Supplement: Supplementary information [file develop-149-200173-s1.pdf]

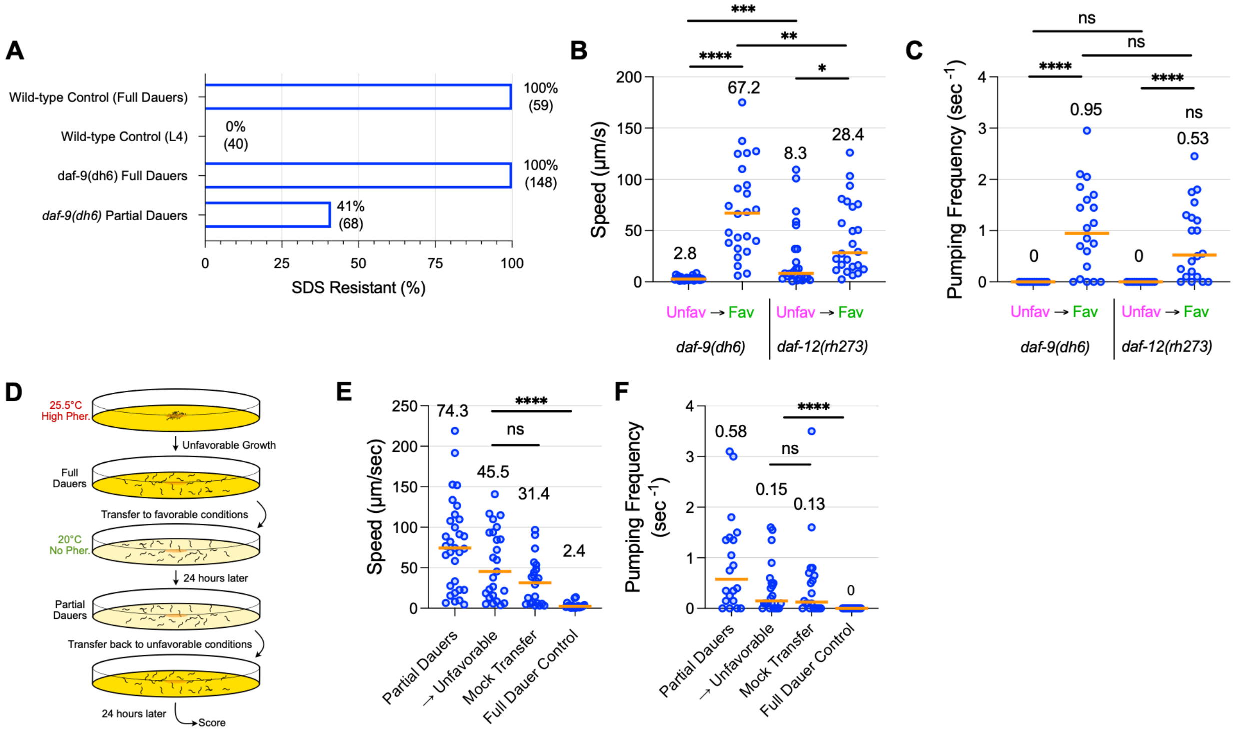

Fig. S1. (A) *daf-9(dh6)* full dauers are completely SDS resistant, while *daf-9(dh6)* partial dauers are SDS sensitive. Also shown are wild-type control animals grown under unfavorable conditions to form full dauers or under favorable conditions to form SDS-sensitive L4 larvae. Displayed are the percentages of animals that survived SDS treatment and the corresponding number of animals treated. (B,C) *daf-12(rh273)* phenocopies the *daf-9(dh6)* partial dauer exit phenotype. The Daf-c allele *daf-12(rh273)* shows a similar partial dauer exit phenotype to *daf-9(dh6)* as measured by locomotion speed and pumping frequency, as *daf-12(rh273)* full dauers formed under unfavorable conditions were induced to become partially exited dauers that (B) moved faster and (C) pumped more frequently following transfer to favorable conditions. Note that *daf-12(rh273)* full dauers tend to be slightly more mobile than N2 or *daf-9(dh6)* dauers, despite being fully pumping quiescent. (D-F) The partial dauer exit phenotype is not reversible. (D) Experimental schematic for reversibility assay. *daf-9(dh6)* partial dauers were produced by first inducing full dauers under unfavorable growth conditions followed by transfer to favorable conditions for 24 hours (Partial Dauers). Partially exited dauers were then transferred back to unfavorable conditions for a further 24 hours (→Unfavorable) and assessed for full dauer characteristics, including locomotion and

pumping frequency. A 24-hour incubation under unfavorable conditions did not significantly decrease (E) locomotion speed nor (F) pumping quiescence compared to a mock transfer control, in which partially exited dauers were transferred to favorable conditions for 24 hours. ns, not significant. *, $p<0.05$. ***, $p<0.01$. ****, $p<0.001$ by Mann Whitney test. Each dot is one animal. n ≥ 12 animals for each experimental sample.

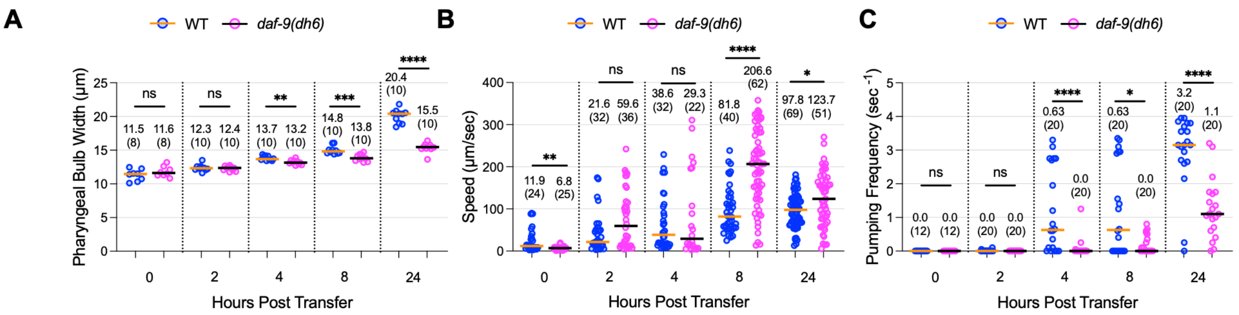

Fig. S2. Comparison of *daf-9(dh6)* dauer exit with wild-type dauer exit. Wild-type dauers and *daf-9(dh6)* full dauers were obtained by growth under unfavorable conditions and then transferred to favorable conditions to induce dauer exit. Animals were scored for (A) speed, (B) pumping frequency, and (C) pharyngeal terminal bulb width before transfer (0 hours post transfer) and at various intervals following transfer. Animals at 0 hours post transfer are identical to "Full Dauers" described elsewhere in the paper, while *daf-9(dh6)* mutants at 24 hours post transfer are identical to "Partial Dauers" obtained through favorable transfer as described elsewhere in the paper. The statistically significant difference between wild-type and *daf-9(dh6)* full dauers at 0 hours post transfer was not consistently observed and occur here because wild-type dauers sporadically show bursts of movement. ns, not significant. *, $p<0.05$. **, $p<0.01$. ***, $p<0.001$. ****, $p<0.0001$ by Mann Whitney test. Horizontal bars and in-graph numbers show median values. Numbers in parentheses indicate number of animals. Each dot is one animal.

Development • Supplementary information

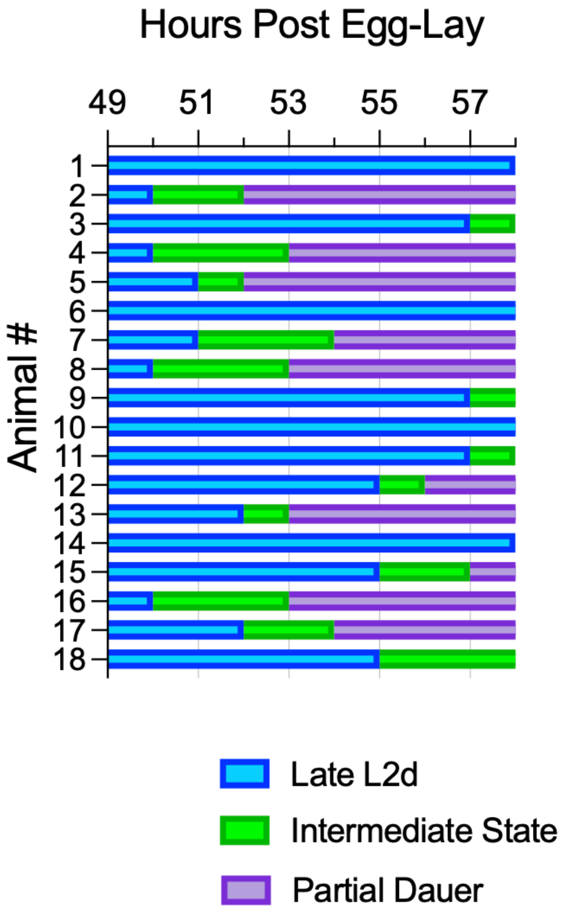

Fig. S3. *daf-9(dh6)* mutants were not observed to enter a full dauer state at lower temperatures. *daf-9(dh6)* worms were grown in absence of exogenously added pheromone at 20.0°C. At 49 hours post egg-lay, animals were individually transferred to new plates and observed every hour. Animals could not be found to pass through a full dauer state as they did at 25.5°C. Instead, following the L2d molt, they passed through an intermediate state that showed characteristics between those of an L2d and a partial dauer larva (see Main Text).

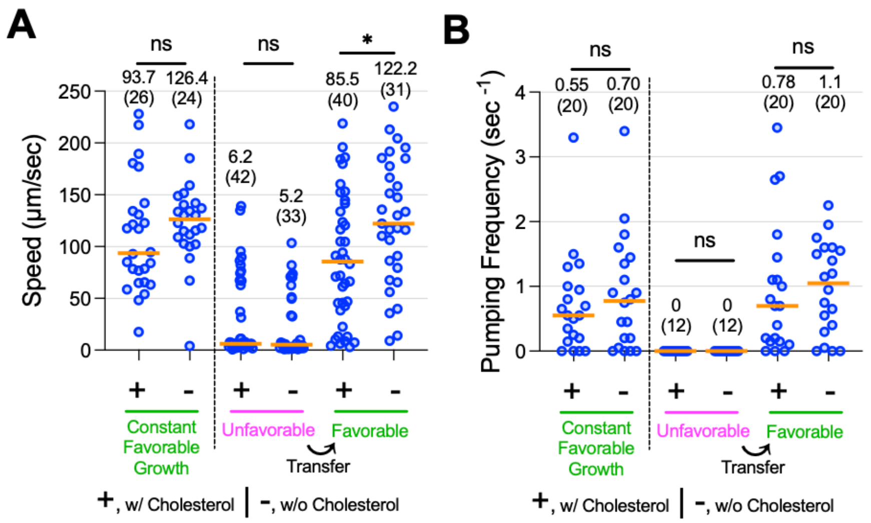

Fig. S4. Effects of cholesterol on the partial dauer phenotype. *daf-9(dh6)* partial dauers were formed by constant growth under favorable conditions, or by first inducing full dauers under unfavorable conditions before transferring to favorable conditions to induce dauer exit. In either case, omission of cholesterol from the growth media does not hinder the formation of partial dauers as measured by (A) speed or (B) pumping frequency. ns, not significant. *, p<0.05, ****,p<0.0001 by Mann Whitney test. Horizontal bars and in-graph numbers show median values. Numbers in parentheses indicate number of animals. Each dot is one animal.

Development • Supplementary information

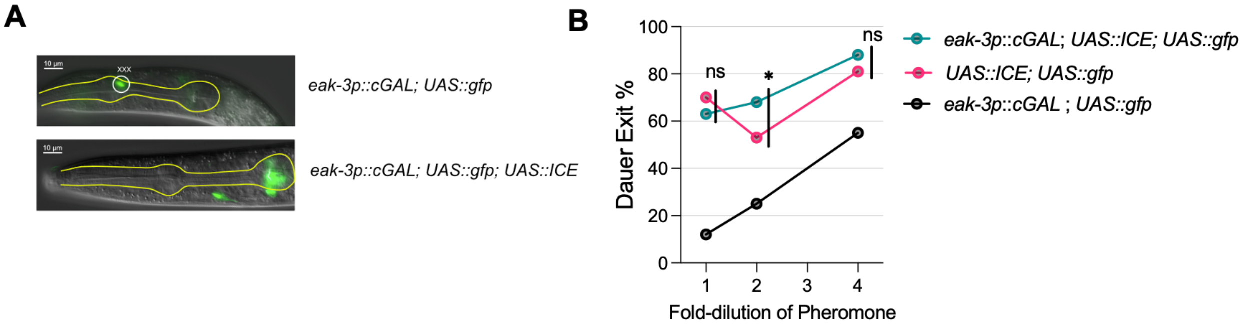

Fig. S5. Genetic ablation of the XXX neuroendocrine cells using the human caspase ICE gene. (A) A transgenic strain using the cGAL bipartite gene expression system expresses *UAS::gfp* using the cell-specific driver *eak-3p::cGAL* in the XXX neuroendocrine cells. The XXX cells are located near the anterior bulb of the pharynx. Expression of the human caspase gene *ICE* using the same XXX-specific *eak-3* cGAL driver shows a loss of fluorescence in the XXX cells. The labeled neuron in the bottom image comes from RFP bleed-through from a co-injection marker labeling the AIY neuron, and the GFP signal in the posterior pharynx is nonspecific expression inconsistently observed in strains bearing the *UAS::gfp* transgene. (B) Genetic ablation of the XXX cells does not drastically impact dauer exit. Dauers whose XXX cells were genetically ablated were transferred to plates with decreasing pheromone concentrations and scored for dauer exit 24 hours later. Note that animals bearing the integrated *UAS::ICE* transgene formed SDS-sensitive dauers that exited at higher rates than the cGAL driver strain (see Materials and Methods). n ≥ 102 animals for each sample. ns, not significant. *, $p<0.05$ by bootstrapped permutation test for a proportion using 10^4 samples.

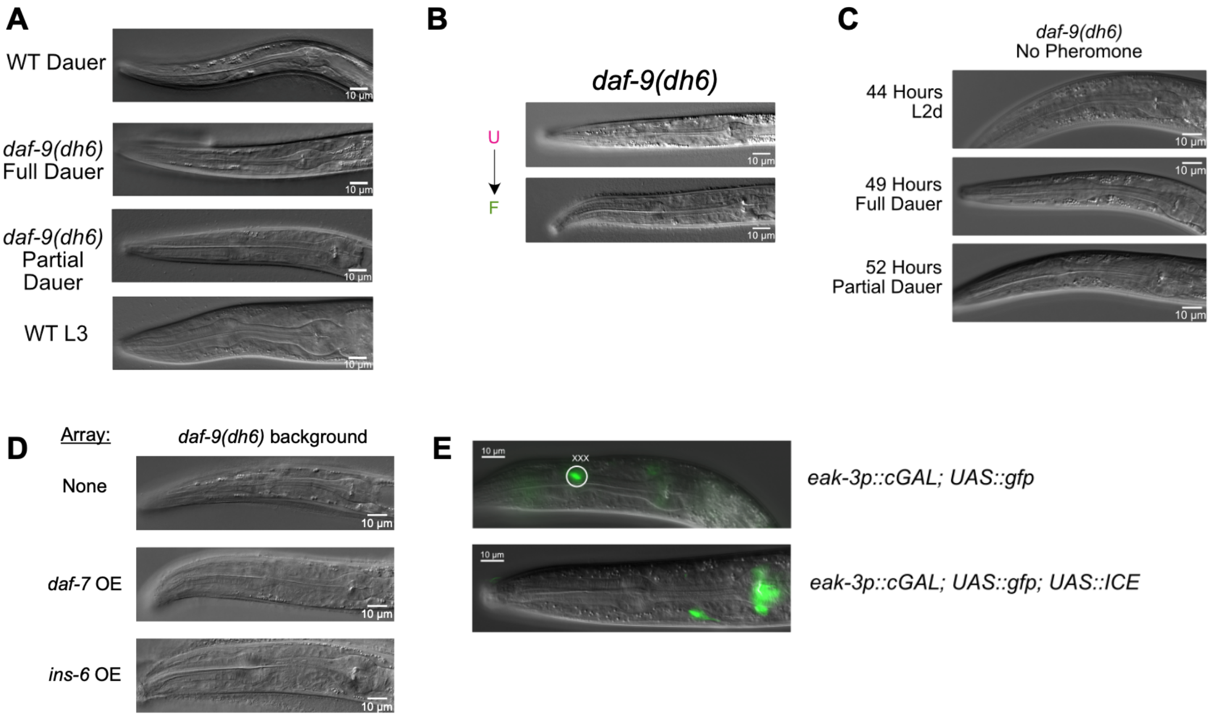

Fig. S6. DIC images of pharynxes shown in (A) Figure 2A, (B) Figure 3G, (C) Figure s4B, (D) Figure 5F, and (E) Figure S5A but with the pharyngeal outlines omitted.

Table S1. Strain names, genotypes, and origins

Click here to download Table S1

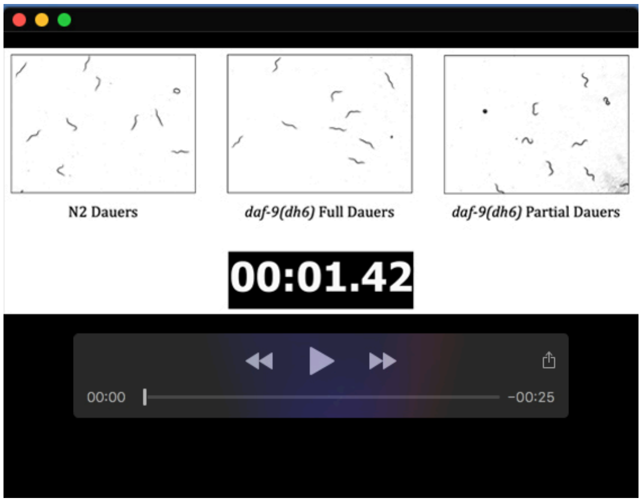

Movie 1. *daf-9(dh6)* full dauers resemble wild-type dauers in terms of locomotion. Shown are 1-minute video recordings of N2 (wild-type) and *daf-9(dh6)* dauers formed under unfavorable growth conditions in comparison to *daf-9(dh6)* partial dauers formed under favorable growth conditions. N2 (wild-type) and *daf-9(dh6)* worms move much more slowly, if at all, compared to *daf-9(dh6)* partial dauers.

Development • Supplementary information

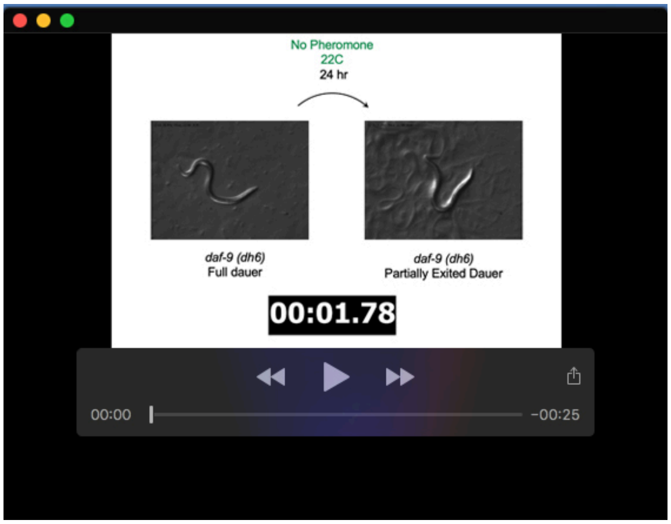

Movie 2. Locomotion behavior of *daf-9(dh6)* partially exited dauers. Shown are ten-second recordings of (left) a *daf-9(dh6)* full dauer formed under unfavorable growth conditions and (right) a *daf-9(dh6)* partially exited dauer formed by transferring full *daf-9(dh6)* dauers from unfavorable to favorable conditions for 24 hours. A second partially exited dauer can be seen crawling at high speed in the background midway through the recording. The *daf-9(dh6)* partially exited dauer performs many small head movements and frequently reverses, unlike *daf-9(dh6)* full dauers which remain idle.
