# Supplementary material for: Both entry to and exit from diapause arrest in Caenorhabditis elegans are regulated by a steroid hormone pathway
Source: Development. 2022 May 3;149(9):dev200173. doi: 10.1242/dev.200173 (PMC9148571; doi:10.1242/dev.200173)
Supplement: Reviewer comments [file develop-149-200173_review_history.pdf]

# Both entry to and exit from diapause arrest in *Caenorhabditis elegans* are regulated by a steroid hormone pathway

Mark G. Zhang and Paul W. Sternberg
DOI: 10.1242/dev.200173

Editor: Swathi Arur

Review timeline

Original submission:	6 September 2021
Editorial decision:	25 October 2021
First revision received:	25 January 2022
Editorial decision:	2 March 2022
Second revision received:	22 March 2022
Accepted:	24 March 2022

Original submission

First decision letter

MS ID#: DEVELOP/2021/200173

MS TITLE: Both entry to and exit from diapause arrest in *Caenorhabditis elegans* are regulated by a steroid hormone pathway

AUTHORS: Mark Guangde Zhang and Paul Warren Sternberg

I have now received all the referees reports on the above manuscript, and have reached a decision. All three reviewers agree on the rigor of the work conducted and while there is some disagreement on impact to developmental biologists in general, I believe the work is careful and important for the field and we would like to publish a revised manuscript in Development, provided that the referees' comments can be satisfactorily addressed. Reviewer 3 especially provides recommendations which I believe will greatly increase the rigor of analysis and clarity of the study. Please attend to all of the reviewers' comments in your revised manuscript and detail them in your point-by-point response. If you do not agree with any of their criticisms or suggestions explain clearly why this is so.

Reviewer 1

Advance summary and potential significance to field

The authors find the role of the DAF-9 Cytochrome P450 in recovery from dauer is similar to its role in entry to dauer: synthesis of steroid hormone is required for progression to reproductive development.

Comments for the author

Zhang and Sternberg investigate a dual puzzle in the molecular genetic control of dauer formation and exit from the dauer state: why disruption of the DAF-9 P450 enzyme confers a partial dauer phenotype and how dauer recovery is regulated. The latter has proved difficult to study because most mutations conferring a dauer constitutive (Daf-c) phenotype also somewhat regulate recovery.

© 2022. Published by The Company of Biologists under the terms of the Creative Commons Attribution License (https://creativecommons.org/licenses/by/4.0/).

(An exception is the daf-28(sa191) mutation, which triggers proteostatic stress in the ASJ neuron, which apparently induces dauer formation but permits rapid recovery.) The authors use a series of elegant approaches to determine that the daf-9 mutant animals form dauer that then partially recover, but that cannot fully recover. Unfavorable growth conditions (high temperature plus dauer-inducing pheromone) or mutation of upstream InsR and TGF-beta signals block recovery of the daf-9 mutant, arguing that signals promoting reproductive development somehow function in parallel to DAF-9.

The manuscript is well written, experiments were rigorously performed and generated enthusiasm for the incisive approach to the question at hand. What concerns this reviewer is that this work provides incremental advances and corroboration of long-standing hypotheses and extension of existing findings for DAF-9. For example, the requirement of DAF-9 for reproductive development upon entry into the dauer state is recapitulated upon exit of the dauer state. And so while a small puzzle in the dauer question has been answered, it is not clear that these findings will be of broad interest to the developmental community, but rather is relevant only to a small group of specialists. As such, I am reluctant to recommend Development as a target journal.

Minor comments:

1) Line 139: "steroid hormone gene" is a vague formulation
2) Are sentences in lines 142-146 reversed?
3) Use "WT" rather than "N2" in figures: less jargon
4) Shouldn't L3 animals be used as a non-dauer control for experiments in Figure 2 to provide information on whether "partial dauers" are incompletely penetrant for certain phenotypes? For that matter, shouldn't conventional standards for full dauer and partial dauer be used as benchmarks, e.g. daf-2 dauers and some Daf-C mutation with a daf-16 mutation?
5) Consider using the daf-28(sa191) animals as a positive control for when recovery ensues, which form dauers but recover almost immediately. If the transition to partial dauers in the daf-9 mutants lag behind recovery of sa191 mutants, DAF-9 may be interpreted to delay the trigger of exit . (not necessary, just enthusiastic about the possibilities).
6) Ln 504. Is comparable the right word here? Maybe "developmentally analogous?"

Reviewer 2

Advance summary and potential significance to field

The study of dauer arrest in C. elegans has yielded important insights into how animals exhibit developmental plasticity in the context of changing environmental conditions. The vast majority of work in this field has focused on mechanisms governing dauer entry in response to unfavorable environmental conditions; comparatively little is known about the mechanisms governing dauer exit and resumption of reproductive development in response to improving environmental conditions. In this manuscript the authors address the role of the DAF-9 dafachronic acid (DA) steroid hormone biosynthetic pathway in dauer exit.

It is well established that the DAF-9 DA pathway is required for reproductive development in favorable environments. However, in contrast to loss-of-function mutations that reduce DAF-2 insulin-like or DAF-7 TGF-beta-like signaling, which result in developmental arrest as complete dauers in animals grown under favorable conditions, daf-9 null mutants, in which DA is not detected, arrest as partial dauers when grown in favorable conditions. Through a series of simple yet elegant experiments, the authors reveal that these daf-9 mutant partial dauers are likely post-dauers that are arrested in the process of exiting dauer. The partial dauer phenotype in daf-9 mutants requires DAF-2 and DAF-7 signaling suggesting that favorable conditions promote partial dauer exit in daf-9 mutants by inducing DAF-2 and DAF-7 pathway activity. The daf-9 partial dauer phenotype is rescued by exogenous DA and hypodermal daf-9 overexpression, indicating that DAF-9-dependent DA biosynthesis is necessary for dauer exit. The spatiotemporal regulation of hypodermal DAF-9 expression during dauer exit mirrors its previously described regulation during dauer entry.

© 2022. Published by The Company of Biologists under the terms of the Creative Commons Attribution License (https://creativecommons.org/licenses/by/4.0/).

This work provides a surprising new insight into the role of the DAF-9 DA pathway in dauer exit and the nature of the "partial dauer," a nebulous description that has plagued the field for decades. One important implication from this work is that, whereas DAF-9 and DAF-12 likely act downstream of DAF-2 and DAF-7 pathways in the control of dauer entry, the activity of unliganded DAF-12 alone does not suffice to maintain dauer arrest in the absence of DAF-16 or DAF-3/DAF-5 activity. Thus, interactions among the three major dauer regulatory pathways (DAF-9 DA, DAF-2, and DAF-7) in the control of dauer exit are more complex than previously appreciated. All of the experiments presented are well-designed and well-executed, and the data are convincing.

Comments for the author

There are a few minor issues that should be addressed in a revised manuscript:
1. Line 115: DAF-16 doesn't really control the insulin pathway; it is the main downstream target of the insulin pathway.
2. Lines 377-378: The phrase beginning with "With..." is unclear.
3. Line 467: "...competent for complete dauer exit..."?

Reviewer 3

Advance summary and potential significance to field

This manuscript examines the role of steroid hormones in regulating exit from the dauer diapause state in C. elegans. The manuscript presents a careful examination of partial dauers made by daf-9 mutants that are deficient in dafachronic acid (DA) steroid hormone production, concluding that these partial dauers are dauers that have initiated but failed to complete dauer exit, and that thus, complete exit from dauer requires DA steroid hormones. These major conclusions of the paper are well-supported by the experimental evidence. Convincing experiments demonstrate that daf-9 mutants make full dauers in unfavorable conditions, that these animals then become partial dauers when shifted to favorable conditions, and that many daf-9 partial dauers formed in favorable conditions transit through a transient full dauer state on their way to becoming partial dauers. Finally, it is shown that DA steroid hormones promote exit of arrested daf-9 partial dauers. This is a solid study and my critiques are all relatively minor.

Comments for the author

Minor Points
1. The paper could be clearer on precisely how daf-9 and DA steroid hormones are required for dauer exit. Though it is true that daf-9 and DA are required for “complete” dauer exit as accurately stated in the Abstract, the take-home message of the paper often seems to be that daf-9 and DAs are required for “dauer exit” without getting into the nuance of which aspect of exit (e.g. lines 130-131 and elsewhere). The idea that DA hormones are required for exit is unintuitive and paradoxical given that daf-9 mutants actually initiate dauer exit rapidly under favorable conditions. Though the paper is generally careful in the precise way it talks about exit, I think it would be clearer to explicitly state the precise role of daf-9 and DA in dauer exit as follows. Based on the data in this paper, I would argue that there are two steps to dauer exit, only one of which depends on daf-9 and steroid hormones. First, an animal needs to initiate dauer exit, which leads it from the full dauer state to the partial dauer state. This step does not seem to require DA steroid hormones as daf-9 mutants initiate dauer exit from the full dauer state to the partial dauer state rapidly. Instead, this initial exit is likely regulated in a similar way as dauer entry – it is blocked by high levels of dauer pheromones, or in Daf-c mutants like daf-2 and daf-7. When pheromone is low (“favorable conditions”) initial exit occurs to the partial dauer state in a DA-independent fashion, and then there is a second stage of regulation going from partial dauers to non-dauers. daf-9 and DA steroid hormones are both necessary and sufficient for this second step, exiting from the partial dauer stage and resuming reproductive development. In one sense, it could be argued that daf-9 and DAs are required for full “execution” of dauer exit, not the “decision” to exit (just as daf-16 partial dauers are sometimes suggested to be defective in the execution of dauer formation rather than the decision). So ultimately, the action of DA hormones is required for reproductive development, whether it is to bypass dauer entirely at the L2 stage or whether it is to fully recover after forming dauers.

© 2022. Published by The Company of Biologists under the terms of the Creative Commons Attribution License (https://creativecommons.org/licenses/by/4.0/).

2. Some figures would benefit from non-dauer controls. For example, in Fig 2, it would be nice to see the pharynx width, speed, and pumping frequency of non-dauer L3s as a comparison. Without these controls, it isn't clear which aspects of the daf-9 phenotype are dauer-like vs. non-dauer. Similarly, in Fig 3E, the daf-9 partial dauers have a speed similar to the recovered WT dauers. After 24 hours in favorable conditions, I would think the WT would be at L4 stage (i.e. fully non-dauer), yet this is one piece of evidence that daf-9 full dauers can become partial dauers upon a shift to favorable conditions. So does a partial dauer move at the same speed as an L4? If so, speed would not be a very useful measure in defining an animal as a partial dauer because it doesn't distinguish partial dauers and non-dauers. Including a WT L4 control would be useful. Pumping frequency in 3F is clearly less for daf-9 partial dauers than recovered WT dauers (presumed L4), but what about pharynx width shown in 3G? Is that more like a dauer or a non-dauer? Again, a WT L4 control would be useful.

3. Related to point 2 above, it would be nice to show that WT dauers at early stages of recovery (much less than 24 hours) resemble daf-9 partial dauers. That would demonstrate there is likely nothing abnormal or incomplete about daf-9 partial dauers and really nail the idea that the daf-9 partial dauer phenotype is due to defects in dauer exit rather than dauer formation.

4. Though strong evidence is presented that some daf-9 partial dauers transit through a transient dauer state (Fig. 4), it also seems likely based on the data in Figs 4 and S2 that daf-9 can also go from an L2d to a partial dauer without becoming a full dauer, though this possibility is not discussed. This raises several questions. Are the daf-9 partial dauers formed at 20° without going through a full dauer state (Fig S2) different than those formed at 25.5° (Fig 4)?
If WT L2d are shifted to favorable growth conditions before becoming dauer, do they go through a partial dauer state that resembles daf-9 mutants? Do daf-9 L2d look the same as WT L2d?

5. Related to point 4 above, the presumed daf-9 mutant L2d pharynx shown in Fig 4B looks somewhat slim – a WT L2d pharynx photo would be useful as comparison.

6. It is stated twice (lines 117 and 144) that there is only "anecdotal evidence" that daf-9 and daf-12 mutants form full dauers under unfavorable conditions.
However, the two papers cited (Antebi et al. 1998; Gerisch et al. 2001) both have tables with quantitative data showing full dauers formed in these mutants under starvation conditions. I would suggest to not use the phrase "anecdotal evidence."

7. Lines 332-334: it is suggested that DA does not promote exit of full daf-9 dauers in the presence of pheromone because of inaccessibility of DA. Though this is possible, another possibility is that DA only promotes dauer exit after an initial decision to exit that does not occur in the presence of pheromone (see point 1 above).

8. It appears that the WT and daf-9 full dauer speed data shown in Fig 2C may be the same data shown in Fig 3E. If so, this should be stated explicitly, and any other data reused between different figure panels should be stated. Were all the data in Fig 3E from experiments performed in parallel? If not, this should be stated.

9. It would be helpful to show DIC micrographs of the pharynx without the yellow outline drawn on top since it is very hard to see the pharynx boundaries with the drawn outline (Figs 2A, 3G, 4B, S3). I would recommend a supplementary figure showing the same images without the outline.

10. It is unclear which daf-9 mutant is shown in Fig 3G (presumably dh6 but not stated). Please state this in the legend.

11. It would be helpful to state in the Fig 6C legend that these are WT dauers not daf-9 dauers.

12. I was confused by Fig 7D. It seems that the presence of the UAS::ICE transgene promotes dauer exit, regardless of the presence of the XXX cGAL driver, which seems odd. Then I read in the Methods that dauers with the UAS::ICE transgene are SDS-sensitive. Are these real full dauers or partial dauers? If they aren't normal full dauers, it seems questionable using them in an assay on dauer exit. Perhaps the UAS::ICE integration site or a mutation in that background caused by

© 2022. Published by The Company of Biologists under the terms of the Creative Commons Attribution License (https://creativecommons.org/licenses/by/4.0/).

integration affect the dauer state? Given these concerns, I would recommend cutting this experiment from the paper. The laser ablation experiment in Fig 7C is much cleaner.

13. In Fig S3, it is stated that the labeled neuron is actually due to bleedthrough of RFP in a coinjection marker. This coinjection marker should be listed in the genotype of this strain in Table S1. Also, it appears that there might also be some GFP in the posterior pharynx in this image? Any explanation?

14. Typos: line 204 (“exists” should be exist), line 298 (“partial dauer exit formation” – should just be partial dauer exit), line 378 (“throughout” should be throughput), line 508 (conservation is highlighted), line 657 (the genotype should be heterozygous for the balancer), line 659 (“mutatns”), line 933 (“presence of absence”, should be “or”).
Reviewed (and signed) by Michael Ailion

First revision

Author response to reviewers' comments

Responses to Reviewer 1

Zhang and Sternberg investigate a dual puzzle in the molecular genetic control of dauer formation and exit from the dauer state: why disruption of the DAF-9 P450 enzyme confers a partial dauer phenotype and how dauer recovery is regulated. The latter has proved difficult to study because most mutations conferring a dauer constitutive (Daf-c) phenotype also somewhat regulate recovery. (An exception is the daf-28(sa191) mutation, which triggers proteostatic stress in the ASJ neuron, which apparently induces dauer formation but permits rapid recovery.) The authors use a series of elegant approaches to determine that the daf-9 mutant animals form dauer that then partially recover, but that cannot fully recover. Unfavorable growth conditions (high temperature plus dauer-inducing pheromone) or mutation of upstream InsR and TGF-beta signals block recovery of the daf-9 mutant, arguing that signals promoting reproductive development somehow function in parallel to DAF-9. The manuscript is well written, experiments were rigorously performed and generated enthusiasm for the incisive approach to the question at hand. What concerns this reviewer is that this work provides incremental advances and corroboration of long-standing hypotheses and extension of existing findings for DAF-9. For example, the requirement of DAF-9 for reproductive development upon entry into the dauer state is recapitulated upon exit of the dauer state. And so while a small puzzle in the dauer question has been answered, it is not clear that these findings will be of broad interest to the developmental community, but rather is relevant only to a small group of specialists. As such, I am reluctant to recommend Development as a target journal.

Response: We appreciate that the reviewer finds our manuscript to be well written and the experiments rigorously executed. We would like to address some of the reviewer’s concerns about the breadth and impact of our study as it relates to *Development* being the right fit for our manuscript.

We maintain that this study significantly contributes to our understanding of diapause regulation and will generate interest and citations from the broader developmental biology community, both within and outside the *C. elegans* dauer field. The fact that the requirement of DAF-9 in dauer entry is recapitulated upon exit of the dauer state may not be surprising, but it also should not be taken for granted. Previous studies have shown that there exist important distinctions in how the insulin-like and TGF-β pathways differentially regulate dauer entry and exit (Cornils et al., 2011; Fernandes de Abreu et al., 2014; Ouellet et al., 2008). The fact that DAF-9 function is conserved between entry and exit is therefore an important finding that has broad ramifications for understanding how other animal species regulate entry and exit from diapause.

Our findings concerning the partial dauer puzzle will be of immense interest to the large community that studies the *C. elegans* dauer state, while the comparative analysis of *daf-9*

© 2022. Published by The Company of Biologists under the terms of the Creative Commons Attribution License (https://creativecommons.org/licenses/by/4.0/).

function between dauer entry and exit will be of broad interest to those studying diapause regulation. Our study is among the first to explicitly compare diapause entry and exit at the molecular level, and thus we establish *C. elegans* as a platform to study the precise molecular details of how dauer entry and dauer exit are similarly or differentially regulated. We expect this manuscript to generate enthusiasm and lay groundwork for future studies that pursue a comprehensive understanding of diapause regulation.

Minor comments:
1) Line 139: "steroid hormone gene" is a vague formulation
- We have replaced "steroid hormone gene" with "gene that acts in a steroid hormone pathway".

2) Are sentences in lines 142-146 reversed?
- We have switched the two sentences in those lines for better readability.

3) Use "WT" rather than "N2" in figures: less jargon
- We have replaced all instances of "N2" with "WT" in our figures.

4) Shouldn't L3 animals be used as a non-dauer control for experiments in Figure 2 to provide information on whether "partial dauers" are incompletely penetrant for certain phenotypes? For that matter, shouldn't conventional standards for full dauer and partial dauer be used as benchmarks, e.g. daf-2 dauers and some Daf-C mutation with a daf-16 mutation?
- In Figure 2, we have added a wild-type L3 control for comparison. Figure 2 shows that *daf-9(dh6)* partial dauers have smaller pharynxes and much slower pumping frequencies than that of L3 larvae, but they actually move slightly faster.
- When it comes to conventional standards for full dauers and partial dauers, we use wild-type full dauer controls in Figure 2. These wild-type dauers are induced by high pheromone concentrations, and so we believe them to be more endogenously relevant than Daf-C mutants such as *daf-2* for use as a control. Furthermore, we measure *daf-2* and *daf-7* metrics in Figure 5, and the numbers are comparable to that of wild-type dauers. For partial dauers, it is unclear whether a "conventional standard" truly exists. As mentioned in the discussion, partial dauers formed by Daf-C; *daf-16* double mutants need to be further tested and compared against *daf-9* partial dauers, as it is possible they are distinct types of partial dauers. The analysis presented in this paper specifically applies to dauers formed by steroid hormone pathway mutants such as *daf-9*, *daf-12*, *daf-36*, etc..

5) Consider using the daf-28(sa191) animals as a positive control for when recovery ensues, which form dauers but recover almost immediately. If the transition to partial dauers in the daf-9 mutants lag behind recovery of sa191 mutants, DAF-9 may be interpreted to delay the trigger of exit . (not necessary, just enthusiastic about the possibilities).
- We appreciate the experimental logic presented here, and so we decided to perform a similar experiment that analyzes the temporal progression of dauer exit in *daf-9(dh6)* mutants. We performed a new experiment, shown in Figure S2, in which we compare the progression of dauer exit between wild-type and *daf-9(dh6)* mutant animals. Figure S2 shows that the exit of *daf-9(dh6)* dauers is indeed slower than that of wild-type animals as measured by pharyngeal bulb width and pumping frequency, suggesting that *daf-9* may also be necessary for the correct pace of dauer exit.

6) Ln 504. Is comparable the right word here? Maybe "developmentally analogous?"
- To avoid confusion, we have omitted the word "comparable" altogether.

Responses to Reviewer 2

The study of dauer arrest in C. elegans has yielded important insights into how animals exhibit developmental plasticity in the context of changing environmental conditions. The vast majority of work in this field has focused on mechanisms governing dauer entry in response to unfavorable environmental conditions; comparatively little is known about the mechanisms governing dauer exit and resumption of reproductive development in response to improving environmental conditions. In this manuscript the authors address the role of the DAF-9 dafachronic acid (DA) steroid hormone biosynthetic pathway in dauer exit.

© 2022. Published by The Company of Biologists under the terms of the Creative Commons Attribution License (https://creativecommons.org/licenses/by/4.0/).

It is well established that the DAF-9 DA pathway is required for reproductive development in favorable environments. However, in contrast to loss-of-function mutations that reduce DAF-2 insulin-like or DAF-7 TGF-beta-like signaling, which result in developmental arrest as complete dauers in animals grown under favorable conditions, daf-9 null mutants, in which DA is not detected, arrest as partial dauers when grown in favorable conditions. Through a series of simple yet elegant experiments, the authors reveal that these daf-9 mutant partial dauers are likely post-dauers that are arrested in the process of exiting dauer. The partial dauer phenotype in daf-9 mutants requires DAF-2 and DAF-7 signaling, suggesting that favorable conditions promote partial dauer exit in daf-9 mutants by inducing DAF-2 and DAF-7 pathway activity. The daf-9 partial dauer phenotype is rescued by exogenous DA and hypodermal daf-9 overexpression, indicating that DAF-9-dependent DA biosynthesis is necessary for dauer exit. The spatiotemporal regulation of hypodermal DAF-9 expression during dauer exit mirrors its previously described regulation during dauer entry.

This work provides a surprising new insight into the role of the DAF-9 DA pathway in dauer exit and the nature of the "partial dauer," a nebulous description that has plagued the field for decades. One important implication from this work is that, whereas DAF-9 and DAF-12 likely act downstream of DAF-2 and DAF-7 pathways in the control of dauer entry, the activity of unliganded DAF-12 alone does not suffice to maintain dauer arrest in the absence of DAF-16 or DAF-3/DAF-5 activity. Thus, interactions among the three major dauer regulatory pathways (DAF-9 DA, DAF-2, and DAF-7) in the control of dauer exit are more complex than previously appreciated. All of the experiments presented are well-designed and well-executed, and the data are convincing.

Response: We are pleased to hear that this reviewer finds our work to be interesting and the experiments properly performed. We especially appreciate the reviewer’s note about the activity of unliganded DAF-12 being insufficient to maintain a full dauer state, as we find it to be a concise and insightful interpretation of our data.

Reviewer 2 Comments for the Author...

There are a few minor issues that should be addressed in a revised manuscript:

1. Line 115: DAF-16 doesn't really control the insulin pathway; it is the main downstream target of the insulin pathway.
   - We have rewritten line 115 and replaced “controls the insulin pathway” with “is the major downstream target of the insulin pathway.”

2. Lines 377-378: The phrase beginning with "With..." is unclear.
   - For clarity, we have replaced this phrase with the following: “To validate this finding, we also genetically ablated the XXX cells by expressing the human caspase gene *ICE* from the XXX-specific promoter *eak-3p* using the cGal bipartite expression system for *C. elegans*”

3. Line 467: "...competent for complete dauer exit..."?
   - We have rewritten the sentence to: “We established that steroid hormones are essential for full dauer exit by showing that *daf-9(dh6)* dauers only partially exit in the absence of Δ7-DA but completely exit when supplemented with Δ7-DA at nanomolar concentrations.”

Responses to Reviewer 3

Reviewer 3 Advance Summary and Potential Significance to Field...

This manuscript examines the role of steroid hormones in regulating exit from the dauer diapause state in C. elegans. The manuscript presents a careful examination of partial dauers made by daf-9 mutants that are deficient in dafachronic acid (DA) steroid hormone production, concluding that these partial dauers are dauers that have initiated but failed to complete dauer exit, and that thus, complete exit from dauer requires DA steroid hormones. These major conclusions of the paper are well-supported by the experimental evidence. Convincing experiments demonstrate that daf-9 mutants make full dauers in unfavorable conditions, that these animals then become partial dauers when shifted to favorable conditions, and that many daf-9 partial dauers formed in favorable

© 2022. Published by The Company of Biologists under the terms of the Creative Commons Attribution License (https://creativecommons.org/licenses/by/4.0/).

conditions transit through a transient full dauer state on their way to becoming partial dauers. Finally, it is shown that DA steroid hormones promote exit of arrested daf-9 partial dauers. This is a solid study and my critiques are all relatively minor.

Response: We are happy to hear that this reviewer finds our work to be a convincing study. This reviewer has raised a number of important considerations and suggested experiments, each of which we have accounted for below. We believe that these changes strengthen the claims and overarching narrative of our work.

Reviewer 3 Comments for the Author...

Minor Points

1. The paper could be clearer on precisely how daf-9 and DA steroid hormones are required for dauer exit. Though it is true that daf-9 and DA are required for “complete” dauer exit as accurately stated in the Abstract, the take-home message of the paper often seems to be that daf-9 and DAs are required for “dauer exit” without getting into the nuance of which aspect of exit (e.g. lines 130-131 and elsewhere). The idea that DA hormones are required for exit is unintuitive and paradoxical given that daf-9 mutants actually initiate dauer exit rapidly under favorable conditions. Though the paper is generally careful in the precise way it talks about exit, I think it would be clearer to explicitly state the precise role of daf-9 and DA in dauer exit as follows. Based on the data in this paper, I would argue that there are two steps to dauer exit, only one of which depends on daf-9 and steroid hormones. First, an animal needs to initiate dauer exit, which leads it from the full dauer state to the partial dauer state. This step does not seem to require DA steroid hormones as daf-9 mutants initiate dauer exit from the full dauer state to the partial dauer state rapidly. Instead, this initial exit is likely regulated in a similar way as dauer entry – it is blocked by high levels of dauer pheromones, or in Daf-c mutants like daf-2 and daf-7. When pheromone is low (“favorable conditions”) initial exit occurs to the partial dauer state in a DA-independent fashion, and then there is a second stage of regulation going from partial dauers to non-dauers. daf-9 and DA steroid hormones are both necessary and sufficient for this second step, exiting from the partial dauer stage and resuming reproductive development. In one sense, it could be argued that daf-9 and DAs are required for full “execution” of dauer exit, not the “decision” to exit (just as daf-16 partial dauers are sometimes suggested to be defective in the execution of dauer formation rather than the decision). So ultimately, the action of DA hormones is required for reproductive development, whether it is to bypass dauer entirely at the L2 stage or whether it is to fully recover after forming dauers.

- We agree with the reviewer’s suggested model for our data, and we have even performed an additional experiment showing the sufficiency of the insulin and TGF-beta pathway to induce a partial dauer state (Figure 5C-F), which complements our current loss-of-function studies (Figure 5A-B). These results are in line with a 2-stage model of dauer exit in which the insulin and TGF-beta pathways are both necessary and sufficient for the first stage, while steroid hormones promote the second stage.
- We now explicitly propose this model in the Discussion with the following paragraph: “In summary, our results are consistent with a model in which dauer exit comprises two stages. The first stage involves the transition from a full dauer to a partially exited dauer and is not dependent on *daf-9* but is instead mediated by insulin and TGF-ß signaling (**Fig. 5**), Since insulin and TGF-ß pathway ligand-encoding genes are regulated in response to dauer-specific cues such as pheromone and food levels (Li et al., 2003; Ren et al., 1996), this first stage could be considered a “sensory integration” step in the dauer exit decision. The second stage in this dauer exit model describes the transition from a partially exited dauer to a reproductive L4 larvae and is mediated by the steroid hormone pathway (**Fig. 6**). Because this stage encompasses the important developmental steps that entail escape from diapause into reproduction, it could be considered the “execution” step in the dauer exit decision. Further experiments that manipulate insulin, TGF-ß, and steroid hormone pathway activity in full and partial dauers with temporal precision will help evaluate such a model.”

2. Some figures would benefit from non-dauer controls. For example, in Fig 2, it would be nice to see the pharynx width, speed, and pumping frequency of non-dauer L3s as a comparison. Without

© 2022. Published by The Company of Biologists under the terms of the Creative Commons Attribution License (https://creativecommons.org/licenses/by/4.0/).

these controls, it isn't clear which aspects of the daf-9 phenotype are dauer-like vs. non-dauer. Similarly, in Fig 3E, the daf-9 partial dauers have a speed similar to the recovered WT dauers. After 24 hours in favorable conditions, I would think the WT would be at L4 stage (i.e. fully non-dauer), yet this is one piece of evidence that daf-9 full dauers can become partial dauers upon a shift to favorable conditions. So does a partial dauer move at the same speed as an L4? If so, speed would not be a very useful measure in defining an animal as a partial dauer because it doesn't distinguish partial dauers and non-dauers. Including a WT L4 control would be useful. Pumping frequency in 3F is clearly less for daf-9 partial dauers than recovered WT dauers (presumed L4), but what about pharynx width shown in 3G? Is that more like a dauer or a non-dauer? Again, a WT L4 control would be useful.

- In Figure 2, we have added L3 controls for pharynx width, speed, and pumping frequency. These controls indicates that *daf-9(dh6)* partial dauers have smaller pharynxes and slower pumping frequencies compared to non-dauer L3 larvae, but *daf-9(dh6)* partial dauers actually move slightly faster than L3 larvae. We agree that speed may not distinguish between partial dauers and non-dauer L3 or L4, but we maintain that movement speed is a useful metric to indicate exit out of a full dauer state, which is an analysis that is consistently done throughout this paper.
- See the point below about an additional experiment we performed comparing *daf-9(dh6)* partial dauer exit to wild-type dauer exit.

3. Related to point 2 above, it would be nice to show that WT dauers at early stages of recovery (much less than 24 hours) resemble daf-9 partial dauers. That would demonstrate there is likely nothing abnormal or incomplete about daf-9 partial dauers and really nail the idea that the daf-9 partial dauer phenotype is due to defects in dauer exit rather than dauer formation.

- We have performed an additional experiment, shown in Figure S2, that compares the temporal progression of dauer exit between wild-type animals and *daf-9(dh6)* mutants. These results indicate that WT dauers take roughly over 8 hours to resemble *daf-9* partial dauers in terms of pharyngeal pumping and expansion. (8 hours might seem long, but it is because we use a low-quality food source, heat-killed OP50, for dauer exit, which is significantly slower than if we used live OP50).

4. Though strong evidence is presented that some daf-9 partial dauers transit through a transient dauer state (Fig. 4), it also seems likely based on the data in Figs 4 and S2 that daf-9 can also go from an L2d to a partial dauer without becoming a full dauer, though this possibility is not discussed. This raises several questions. Are the daf-9 partial dauers formed at 20° without going through a full dauer state (Fig S2) different than those formed at 25.5° (Fig 4)? If WT L2d are shifted to favorable growth conditions before becoming dauer, do they go through a partial dauer state that resembles daf-9 mutants? Do daf-9 L2d look the same as WT L2d?

- We have made changes to paragraphs in both the Results and Discussion section to address these points. Specific sentences in the new text that answer these questions are bolded here (but not in the manuscript).
- The rewritten Results section paragraph: "We also performed the above single animal observation experiments under more favorable conditions by lowering the temperature to 20°C. However, under these conditions, we were unable to find any *daf-9(dh6)* larvae that went through a full dauer state, despite making observations every hour (**Fig. S2**). ***daf-9(dh6)* grown under these conditions passed through an L2d stage and L2d molt indistinguishable from that of wild-type L2d larvae and L2d larvae formed by *daf-9(dh6)* mutants grown under unfavorable conditions**. Following the L2d molt, these *daf-9(dh6)* mutants instead passed through an intermediate state that involved both elements of being a dauer (a darkened body) as well as partial dauer (pumping, motility), before becoming well-recognizable partial dauers usually within one hour. These observations suggest that high temperatures facilitate formation of full dauers in *daf-9(dh6)* mutant animals in the absence of exogenously added pheromone."
- The rewritten Discussion section paragraph: "Under completely favorable conditions (i.e., no pheromone and low temperature), *daf-9(dh6)* larvae could not be found in a full dauer state (**Fig. S2**). Following the L2d molt, we were only able to find *daf-9(dh6)* mutants in a transient, intermediate state that looked like a hybrid between an L2d and a partial dauer in terms of morphology and behavior. **We could not observe a similar intermediate state in wild-type animals, which we attempted to do by transferring wild-type L2d larvae**

© 2022. Published by The Company of Biologists under the terms of the Creative Commons Attribution License (https://creativecommons.org/licenses/by/4.0/).

that had committed to becoming dauers from unfavorable to favorable conditions (Schaedel et al., 2012). Instead, these animals passed through a full dauer state (data not shown). **These data suggest that *daf-9(dh6)* mutants skip or fail to enter the full dauer state under favorable conditions.** One possibility for this observation is that constant growth under favorable conditions activates insulin and TGF-β pathways in *daf-9(dh6)* mutants and prevents full dauer formation. Alternatively, *daf-9* may be required for full dauer formation under favorable conditions but not unfavorable conditions. Untangling these possibilities requires a better understanding of the molecular effectors downstream of the insulin, TGF-β, and steroid hormone pathways that are directly responsible for the behavioral and morphological changes associated with full dauer formation."

5. Related to point 4 above, the presumed daf-9 mutant L2d pharynx shown in Fig 4B looks somewhat slim - a WT L2d pharynx photo would be useful as comparison.

- Nearly all of the L2d larvae we scored at 44 hours were late L2d larvae (either molting or close to molting), so their pharynxes were slightly slimmer than a mid-stage L2d. To make this clear, we have changed the labeling in Fig. 4 and other similar figures to "Late L2d"

6. It is stated twice (lines 117 and 144) that there is only "anecdotal evidence" that daf-9 and daf-12 mutants form full dauers under unfavorable conditions. However, the two papers cited (Antebi et al. 1998; Gerisch et al. 2001) both have tables with quantitative data showing full dauers formed in these mutants under starvation conditions. I would suggest to not use the phrase "anecdotal evidence."

- We have removed the phrase "anecdotal" in referring to the studies mentioned. The new text reads as follows: Previous reports indicate that the Daf-c steroid hormone mutants *daf-9(dh6)* and *daf-12(rh273)* form full dauers under unfavorable growth conditions (Antebi et al., 1998; Gerisch et al., 2001). To confirm these findings, we grew *daf-9(dh6)* animals under unfavorable conditions, which involves high temperature (25.5°C) and the presence of dauer-inducing pheromone extract (see Materials and Methods). These unfavorable growth conditions yielded *daf-9(dh6)* dauer larvae that matched the characteristics of full dauers formed by wild-type animals.

7. Lines 332-334: it is suggested that DA does not promote exit of full daf-9 dauers in the presence of pheromone because of inaccessibility of DA. Though this is possible, another possibility is that DA only promotes dauer exit after an initial decision to exit that does not occur in the presence of pheromone (see point 1 above).

- This is an excellent point. We have rewritten those lines and added that possibility. It now reads as such: "We also determined whether Δ7-DA could induce dauer exit of *daf-9(dh6)* mutants in the presence of pheromone. Even at 100 nM Δ7-DA, almost all animals remained full dauers (89.4%, n=284). This could be because Δ7-DA is insufficient to induce dauer exit without the dauer first being exposed to favorable conditions that activate insulin and TGF-β pathways. Another possibility could be that their lack of feeding and/or their thickened cuticle (Cassada and Russell, 1975) preclude access to Δ7-DA."

8. It appears that the WT and daf-9 full dauer speed data shown in Fig 2C may be the same data shown in Fig 3E. If so, this should be stated explicitly, and any other data reused between different figure panels should be stated. Were all the data in Fig 3E from experiments performed in parallel? If not, this should be stated.

- We regret not having made this clear in our initial submission. Since we performed additional L3 controls for Figure 2, we actually reran some experiments such that data in Figure 2 and Figure 3 are nearly completely separate. The only piece of data that is reused between the two figures now is a set of pharyngeal pumping frequencies for wild-type dauers and *daf-9(dh6)* dauers obtained under unfavorable growth conditions, both of which consistently yield 0 pumps/sec without fail. We have now noted the reuse of this data in Figure 2.
- All the data in Figure 3E were indeed performed in parallel.

9. It would be helpful to show DIC micrographs of the pharynx without the yellow outline drawn on top since it is very hard to see the pharynx boundaries with the drawn outline (Figs 2A, 3G, 4B, S3). I would recommend a supplementary figure showing the same images without the outline.

© 2022. Published by The Company of Biologists under the terms of the Creative Commons Attribution License (https://creativecommons.org/licenses/by/4.0/).

- We have added an additional Figure, S6, that includes that DIC images in the Figures mentioned above but without the pharynx outline.

10. It is unclear which daf-9 mutant is shown in Fig 3G (presumably dh6 but not stated). Please state this in the legend.

- This is now clearly stated in both the Figure itself (it is labeled as *daf-9(dh6)*) and in the legend

11. It would be helpful to state in the Fig 6C legend that these are WT dauers, not daf-9 dauers.

- The legend now reads as follows: "Overexpression of *daf-9* from the hypoderm-specific and dauer-specific promoter *col-183p* **in a wild-type background** promotes dauer exit."

12. I was confused by Fig 7D. It seems that the presence of the UAS::ICE transgene promotes dauer exit, regardless of the presence of the XXX cGAL driver, which seems odd. Then I read in the Methods that dauers with the UAS::ICE transgene are SDS-sensitive. Are these real full dauers or partial dauers? If they aren't normal full dauers, it seems questionable using them in an assay on dauer exit. Perhaps the UAS::ICE integration site or a mutation in that background caused by integration affect the dauer state? Given these concerns, I would recommend cutting this experiment from the paper. The laser ablation experiment in Fig 7C is much cleaner.

- The presence of the *UAS::ICE* transgene indeed produces SDS-sensitive dauers that exit dauer at a rate higher than the XXX driver strain does. These dauers are also a bit shorter than normal, but they are otherwise normal dauers (pumping quiescent, idle, thin, etc.). Despite these artifacts and abnormalities, we still believe that it is a valuable experiment that supplements the results from the laser ablation experiment, especially because the loss of XXX GFP signal is convincing. But in recognition of these concerns, we have done the following:
    - Move the genetic ablation experiment to the supplement (now Figure S5)
    - Note the artifacts and concerns in both the main text and figure legend

13. In Fig S3, it is stated that the labeled neuron is actually due to bleedthrough of RFP in a coinjection marker. This coinjection marker should be listed in the genotype of this strain in Table S1. Also, it appears that there might also be some GFP in the posterior pharynx in this image? Any explanation?

- We have updated all the genotypes in Table S1 to include the co-injection markers
- The GFP in the posterior pharynx is nonspecific expression seen in strains with the integrated *UAS::gfp* transgene. We have noted this in the figure legend. Both strains in that figure actually have the posterior pharyngeal GFP signal, but it just happens to be more intense in the bottom image.

14. Typos: line 204 ("exists" should be exist), line 298 ("partial dauer exit formation" – should just be partial dauer exit), line 378 ("throughout" should be throughput), line 508 (conservation is highlighted), line 657 (the genotype should be heterozygous for the balancer), line 659 ("mutatns"), line 933 ("presence of absence", should be "or").

- We have fixed each of these typos.

Reviewed (and signed) by Michael Ailion

© 2022. Published by The Company of Biologists under the terms of the Creative Commons Attribution License (https://creativecommons.org/licenses/by/4.0/).

Second decision letter

MS ID#: DEVELOP/2021/200173

MS TITLE: Both entry to and exit from diapause arrest in *Caenorhabditis elegans* are regulated by a steroid hormone pathway

AUTHORS: Mark Guangde Zhang and Paul Warren Sternberg

I have now received all the referees reports on the above manuscript, and have reached a decision. The overall evaluation is positive and we would like to publish a revised manuscript in Development. However, before I can do so, please address Reviewer 2's comments which are textual in nature. If you do not agree with any of their criticisms or suggestions explain clearly why this is so. I do not expect to send this back to the reviewers, thus, please indicate clearly in the revised manuscript the nature of the edits.

Reviewer 1

Advance summary and potential significance to field

The authors provide interesting mechanistic exploration of the recovery process in the C. elegans dauer developmental decision. This process has historically been difficult to address, but this study makes significant inroads into the question.

Comments for the author

A combination of the author's responses, additional data and commentary from other reviewers has convinced me that this manuscript is suitable for publication.

Reviewer 3

Advance summary and potential significance to field

See what I wrote in my review of the original submission. I'm only filling this out because it won't let me submit my review otherwise. I keep getting an error message: "Please provide all of the required recommendations. The following piece is missing: Summary of the advance made in this paper."

Comments for the author

The authors have responded very well to the critiques. The paper has been significantly improved by a number of textual changes as well as a few additional experiments. The new experiment showing sufficiency of insulin pathway activity for the daf-9 partial dauer phenotype is especially interesting. Only a few very minor issues remain.

Minor Points

1. line 226: "*daf-9(dh6)* partially exited dauers move at higher speeds than wild-type dauers do after four hours." The data in Fig S2B show no difference at 4 hours, but higher speed of daf-9 at 8 hours.

2. line 243: "By 49 hours, around 25% of *daf-9(dh6)* mutants grown in the absence of pheromone could be scored as full dauers." In the data in Fig 4B, it looks like about 50% of daf-9 mutants are full dauers.

3. line 452: "When Δ7-DA is supplemented to these partial dauers, full dauer exit occurs (Figure 6B)." This implies that the effect of Δ7-DA was on partial dauers, but in the experiment in Fig 6B, Δ7-DA is actually given to full dauers, and it's unclear if it is acting on full or partial dauers to

© 2022. Published by The Company of Biologists under the terms of the Creative Commons Attribution License (https://creativecommons.org/licenses/by/4.0/).

stimulate dauer exit. More complex experiments would be required to determine the exact stage Δ7-DA is acting, such as exposing full daf-9 dauers to Δ7-DA and then shifting them off Δ7-DA as soon as they initiate recovery, or exposing daf-9 partial dauers to Δ7-DA only after they have? become partial dauers.

Reviewed (and signed) by Michael Ailion

Second revision

Author response to reviewers' comments

Reviewer responses

There were no further suggested edits to the manuscript by Reviewers 1 and 2, and therefore we have only made changes to the manuscript in response to Reviewer 3.

Responses to Reviewer 3

The authors have responded very well to the critiques. The paper has been significantly improved by a number of textual changes as well as a few additional experiments. The new experiment showing sufficiency of insulin pathway activity for the daf-9 partial dauer phenotype is especially interesting.

Only a few very minor issues remain.
Minor Points
1. line 226: “daf-9(dh6) partially exited dauers move at higher speeds than wild-type dauers do after four hours.” The data in Fig S2B show no difference at 4 hours, but higher speed of daf-9 at 8 hours.

Response: We have edited that sentence (changes in bold) to: “daf-9(dh6) partially exited dauers move at higher speeds than wild-type dauers do after eight hours.”

2. line 243: “By 49 hours, around 25% of daf-9(dh6) mutants grown in the absence of pheromone could be scored as full dauers.” In the data in Fig 4B, it looks like about 50% of daf-9 mutants are full dauers.

Response: We have edited that sentence (changes in bold) to: “By 49 hours, around 50% of daf-9(dh6) mutants grown in the absence of pheromone could be scored as full dauers.”

3. line 452: “When Δ7-DA is supplemented to these partial dauers, full dauer exit occurs (Figure 6B).” This implies that the effect of Δ7-DA was on partial dauers, but in the experiment in Fig 6B, Δ7-DA is actually given to full dauers, and it’s unclear if it is acting on full or partial dauers to stimulate dauer exit. More complex experiments would be required to determine the exact stage Δ7-DA is acting, such as exposing full daf-9 dauers to Δ7-DA and then shifting them off Δ7-DA as soon as they initiate recovery, or exposing daf-9 partial dauers to Δ7-DA only after they have become partial dauers.

Response: We have performed the latter experiment suggested by the reviewer (“exposing daf-9 partial dauers to Δ7-DA only after they have become partial dauers”) and added the following to the Results section in line 335: “We also found that we could induce complete dauer exit in daf 9(dh6) partial dauers, obtained by exposing full dauers to favorable conditions for 24 hours, via incubation with 100 nM Δ7-DA (78% become gravid adults within 2 days, n=346).”

We also removed the specific mention to Figure 6B in line 452 to avoid confusion as the reviewer points out.

© 2022. Published by The Company of Biologists under the terms of the Creative Commons Attribution License (https://creativecommons.org/licenses/by/4.0/).

Third decision letter

MS ID#: DEVELOP/2021/200173

MS TITLE: Both entry to and exit from diapause arrest in *Caenorhabditis elegans* are regulated by a steroid hormone pathway

AUTHORS: Mark Guangde Zhang and Paul Warren Sternberg
ARTICLE TYPE: Research Article

I am happy to tell you that your manuscript has been accepted for publication in Development, pending our standard ethics checks.

© 2022. Published by The Company of Biologists under the terms of the Creative Commons Attribution License (https://creativecommons.org/licenses/by/4.0/).
